# Highly Conserved Evolution of Aquaporin PIPs and TIPs Confers Their Crucial Contribution to Flowering Process in Plants

**DOI:** 10.3389/fpls.2021.761713

**Published:** 2022-01-04

**Authors:** Qi Li, Tao Tong, Wei Jiang, Jianhui Cheng, Fenglin Deng, Xiaojian Wu, Zhong-Hua Chen, Younan Ouyang, Fanrong Zeng

**Affiliations:** ^1^Institute of Crop Science, Zhejiang University, Hangzhou, China; ^2^Collaborative Innovation Center for Grain Industry, College of Agriculture, Yangtze University, Jingzhou, China; ^3^Zhejiang Academy of Agricultural Sciences, Hangzhou, China; ^4^School of Science, Western Sydney University, Penrith, NSW, Australia; ^5^China National Rice Research Institute, Hangzhou, China

**Keywords:** aquaporins, evolutionary conservation, flowering, transcriptome, absolute quantification, lodicules, petals

## Abstract

Flowering is the key process for the sexual reproduction in seed plants. In gramineous crops, the process of flowering, which includes the actions of both glume opening and glume closing, is directly driven by the swelling and withering of lodicules due to the water flow into and out of lodicule cells. All these processes are considered to be controlled by aquaporins, which are the essential transmembrane proteins that facilitate the transport of water and other small molecules across the biological membranes. In the present study, the evolution of aquaporins and their contribution to flowering process in plants were investigated *via* an integration of genome-wide analysis and gene expression profiling. Across the barley genome, we found that *HvTIP1;1*, *HvTIP1;2*, *HvTIP2;3*, and *HvPIP2;1* were the predominant aquaporin genes in lodicules and significantly upregulated in responding to glume opening and closing, suggesting the importance of them in the flowering process of barley. Likewise, the putative homologs of the above four aquaporin genes were also abundantly expressed in lodicules of the other monocots like rice and maize and in petals of eudicots like cotton, tobacco, and tomato. Furthermore, all of them were mostly upregulated in responding to the process of floret opening, indicating a conserved function of these aquaporin proteins in plant flowering. The phylogenetic analysis based on the OneKP database revealed that the homologs of TIP1;1, TIP1;2, TIP2;3, and PIP2;1 were highly conserved during the evolution, especially in the angiosperm species, in line with their conserved function in controlling the flowering process. Taken together, it could be concluded that the highly evolutionary conservation of TIP1;1, TIP1;2, TIP2;3 and PIP2;1 plays important roles in the flowering process for both monocots and eudicots.

## Introduction

Anthesis is the key process for the sexual reproduction in flowering plants. Normally, expansion of petals leads to anthesis in eudicots. In contrast to eudicots, the gramineous florets contain different floral organs: two glumes (lemma and palea), two lodicules, three to six stamens, and one carpel from outside to inside, which make their anthesis include two actions: glume opening and glume closing. In cereal crops, the action of glume opening and closing possesses great utilization values for the agricultural production. On the one hand, cleistogamy (glumes fail to open) is a favorable trait to reduce the interaction between self-pollination crops (such as rice, wheat, and barley, etc.) and the environment, which could effectively not only avoid the failure of pollinating caused by pests or adverse environmental conditions during anthesis (Hori et al., 2006; Dahleen et al., 2012) but also prevent transgenes from spreading from genetically modified (GM) crops into wild relatives by reducing their outcrossing rate (Daniell, 2002; Ma and Wang, 2004). On the other hand, glume opening is the basis of cross-pollination to utilize heterosis in crossbreeding of cereal crops (Abdel-Ghani et al., 2004; Zhang et al., 2016), which has made a great contribution to improving crop yield, quality, and tolerance to meet the demand of the increasing global population in the last few decades (Yu et al., 2020; Zhang et al., 2021). In rice production, for instance, the use of hybrid seeds has raised the average rice grain yield by 15–30% compared with the traditional breeding methods, and the super hybrid technology by performing interspecies crossings (indica/japonica) has brought a further advance in rice grain yield of approximately 20% relative to the conventional hybrids (Yuan, 2003; Mondo et al., 2016a). To make better use of hybrid technology in crop production, the main challenge is to ensure the efficient production of hybrid seeds, which is highly restricted by the variation in flowering synchrony of parental lines (Mondo et al., 2016b).

Lodicules are the two diminutive organs analogous to eudicot petals, lying between glumes and the ovary basis in the grass floret (Yoshida, 2012; Zhang et al., 2019). It has been reported that the swelling and withering of lodicules, which are caused by the osmotic potential-forced water accumulation and loss (Liu et al., 2017), play the critical role in the glume opening and closing of gramineous floret. Inter- or intracellular water flow is directly associated with aquaporin proteins (AQPs) localized at the membranes (Li G. et al., 2014). In plants, AQPs are membrane channels belonging to the membrane intrinsic proteins (MIPs) family, which is classified into seven subfamilies in terrestrial plants, including plasma membrane intrinsic proteins (PIPs), tonoplast intrinsic proteins (TIPs), nodulin 26-like intrinsic protein (NIPs), small basic intrinsic proteins (SIPs), the X intrinsic proteins (XIPs), the hybrid intrinsic proteins (HIPs), and the GlpF-like intrinsic proteins (GIPs). The eudicots normally have five subfamilies, including PIPs, TIPs, NIPs, SIPs, and XIPs, whereas monocots lost the XIPs family (Danielson and Johanson, 2008). The GIPs and HIPs only occur in lower non-vascular plant species, such as *Physcomitrella patens* and *Selaginella moellendorffii*, and are lost in higher plants during the evolution (Danielson and Johanson, 2008; Anderberg et al., 2012; Deshmukh et al., 2015). The MIP family plays the essential roles in plant growth and development by facilitating the effective transport of water and some small neutral solutes across all kinds of biological membranes (Verdoucq and Maurel, 2018), including glycerol (Biela et al., 2010), CO_2_ (Uehlein et al., 2003), H_2_O_2_ (Bienert and Chaumont, 2014), and metalloids, such as boron, silicon, and arsenite (Mitani-Ueno et al., 2011; Deng et al., 2020). Moreover, AQPs have been demonstrated to be involved in plant flowering. It was reported that the mutation of *NtPIP2* in tobacco resulted in a delayed anther dehiscence and slowed dehydration (Bots et al., 2005). In rose (*Rosa hybrid*), *PIP2;1* was observed to be significantly expressed in the petal epidermal cells and played a significant role in the expansion of petals and flowering (Ma et al., 2008). Likewise, Kong et al. (2018) found that the transcripts of *PIP* and *TIP* subfamily members were highly abundant during flower opening stages in carnation (*Dianthus caryophyllus*). Recently, the constitutive overexpression of the poplar root-specific *AQP* gene *PtoPIP1;1* in *Arabidopsis* has been reported to accelerate the flowering process of the transgenic lines (Leng et al., 2021). All these pieces of research suggest the importance of AQPs in plant flowering. It can be hypothesized that the changes in the expression of *AQP* genes might regulate the flowering process in crops, which thereby influences the flowering synchrony of parental lines and finally enhances the success in the production of hybrid seeds. However, the function of AQPs in regulating the glume opening and closing in gramineous crops is rarely understood.

Barley (*Hordeum vulgare* L.) is the fourth most important cereal crop with the global production of at least 140 million tons per year (FAOSTAT^1^) (Saisho and Takeda, 2011). Most cultivated barley varieties are maintained by self-pollination with the indehiscent spikelet, which largely restricts the improvement of grain yield and quality in barley by utilizing heterosis (Nair et al., 2010). So far, however, very few efforts have been made to fully understand the mechanism of glume opening and closing in barley. In the present study, 38 HvAQP proteins, including 16 PIPs, 9 TIPs, 11 NIPs, and 2 SIPs, were identified within the whole barley genome, and their gene expression profiling in lodicules during the flowering process was elucidated. Furthermore, the evolution of aquaporins across the entire plant kingdom and their contribution to flowering process in both monocot and eudicot species were also investigated. All these investigations aimed to answer the following questions: Are the AQP proteins functionally conserved during the flowering process in plants? If yes, is the conserved function of AQP proteins in plant flowering linked with their evolutionary conservation?

## Materials and Methods

### Plant Material and Growth Condition

Barley variety “Hu 1154” used in the present study was obtained from Yangzhou University (Yangzhou, China). The well-germinated barley seeds were sown in the soil and grown in a well-controlled growth chamber with the photoperiod of 14-h light/10-h dark, temperature of 23^°^C light/18^°^C dark, light intensity of 200 ± 25 μmol⋅m^–2^⋅s^–1^, and relative humidity of 65% according to our previous experience in the studies on barley (Cai et al., 2019). At the anthesis stage, different barley tissues were collected for gene expression profiling.

To compare the gene expression of AQPs in different plant species, seeds of barley (var. Hu 1154), coupled with the other five typical plant species, including rice (var. Nipponbare), maize (inbred line B73), cotton (acc. TM-1), tobacco (var. yun87), and tomato (var. Heinz 1706), were sown in the field contained with sandy loam soil in November 2019 and April 2020, respectively. The soil was fertilized with urea (300-675 kg/hm^2^), calcium superphosphate (375-750 kg/hm^2^), and potassium sulfate (180–360 kg/hm^2^) according to the local guidelines of agricultural production for each crop and watered to field capacity during the growth period. In addition, weeds, insects, and diseases were chemically controlled as required. At the anthesis stage, the lodicules of barley, rice, and maize, as well as the petals of cotton, tobacco, and tomato, were collected to investigate absolute gene expression levels, respectively.

### Identification of the *HvAQPs* Gene Family and Phylogenetic Analysis

*Hordeum vulgare* genome and protein sequences were downloaded from Ensemble plants.^2^ The MIP domain (PF00230) from Pfam database^3^ was used as the query to search the *AQP* genes from the barley genome database. The programs hmmbuild and hmmsearch from the HMMER v3 suite were taken to identity putative *HvAQP* family members, combining with relevant Hidden Markov model (HMM). The new barley-specific HMM was used to search for all members in all barley proteins with an E-value lower than 0.001. After removing all redundant sequences, the conserved MIP domain in the remaining putative protein sequences was examined and confirmed by using CDD,^4^ Pfam, and SMART.^5^ Meanwhile, the transmembrane domain (TMD) of AQPs was predicted using TMHMM Server v.2.0.^6^ Moreover, the molecular weight (MW) and theoretical pI of these proteins were analyzed using online program ProtParam.^7^

The subcellular localization was predicted by Plant-mPloc server^8^ (Abdul Kayum et al., 2018). Finally, all of the non-redundant and high-confidence sequences with a complete MIP domain were preserved and named based on their sequence homology, the annotation from the Uniprot protein database,^9^ and phylogenetic analyses among barley, rice, and *Arabidopsis.*

To clarify the evolutionary relationship of AQP families, amino acid sequences of AQPs from *Arabidopsis* and rice were used to construct a phylogenetic tree aligned by clustalW with the default parameters. Then, the unrooted phylogenetic tree of multiple species was performed by MEGA7.0 software using the Maximum Likelihood (ML) method.

### Gene Structure and Conserved Motif Characterization

To identify the conserved motifs in AQPs, the amino acid sequences of AQP proteins were analyzed using MEME program.^10^ The exon/intron structures of *AQPs* were drawn using GSDS 2.041 (Guo et al., 2007). The HMMER web server (Finn et al., 2015) was employed to identify conserved domains of AQPs. In addition, NPA motifs and transmembrane domains were presented alone using multiple alignment software MEGA version 7.0.

### Chromosomal Location and Duplication

According to the barley gene annotation file, the chromosome position information of the *HvAQPs* was obtained; then, their position and relative distance on the chromosome were displayed by Mapchart software and rendered by Adobe Illustrator CS6 software.

Moreover, the Multiple Collinearity Scan toolkit (MCScanX) software with default parameters was performed for syntenic analysis. In order to display the segmental duplications situation of the putative paralogs *MIP* genes acquired from the barley, the syntenic analysis map was constructed using the based circus tool of TBtools (Chen C. et al., 2020). The inside lines were used to link the putative duplicated genes, and the all-vs-all protein sequence comparisons were employed to implement the document required for MCScanX using DIAMOND v0.8.25.

### RNA Extraction, cDNA Synthesis, RNA-seq, and Real-Time Quantitative PCR Analysis

To investigate the *HvAQP* genes expression level in different tissues of barley, eight tissues, including roots, stems, leaves, rachises, glumes, anthers, pistils, and lodicules, were collected at the anthesis stage. On the other hand, to investigate the expression level of *HvAQP* genes in response to the process of flowering in barley, fresh lodicules at six stages of glume opening and closing were collected, with 50 pairs of lodicules being mixed as one biological replicate. For each tissue or flowering stage, three biological replicates were measured. Total RNA was extracted from either the fresh tissues or lodicules using MiniBEST Plant RNA Extraction Kit (TaKaRa, Cat#9769). RNA quantity and purity were firstly tested by gel electrophoresis and subsequently examined by Bioanalyzer 2100 and RNA 6000 Nano LabChip Kit (Agilent, CA, United States) with RIN number > 7.0. A total amount of 3-μg RNA per sample was used as input material for the RNA sample preparations. Sequencing libraries were generated using NEBNext^®^ Ultra™ RNA Library Prep Kit for Illumina^®^ (NEB, United States), following the manufacturer’s recommendations, and index codes were added to attribute sequences to each sample. The paired-end sequencing was performed using Illumina HiSeq 2500 sequencing platform. Finally, clean reads from the sequencing data were directly used for transcriptome analysis (Yang et al., 2020). With the S1 stage as the control, the genes with | log_2_fold change| ≥ 1 and FDR ≤ 0.05 were considered as differentially expressed genes (DEGs). The Venn diagram and gene ontology analysis of DEGs were performed using the OmicStudio tools^11^ and agriGO platform,^12^ respectively. All of the terms in Gene Ontology (GO) analysis of DEGs are listed in Supplementary Dataset 1. The heatmap based on log_10_-transformed TPM (Transcripts Per Million) values represented the expression levels. A threshold TPM value of 10 was used as a cutoff to define gene expression, and any gene with an expression level of 10 or less was considered either minimally or not expressed (Kröger et al., 2013).

For real-time quantitative PCR (RT-qPCR) validation, the total RNA samples were used to convert to cDNA by using PrimeScript™ RT reagent Kit (TaKaRa, R036A). The expression level of samples was conducted to RT-qPCR analysis on the real-time PCR system (LightCycler^®^ 480 II, 96, software version 1.5, Roche, Switzerland) and SYBR-Green Supermix (Bio-Rad, United States). The reaction was carried out in the following procedures: 95°C for 30 s (95°C, 5 s; 60°C, 30 s), forty cycles, from 60 to 95°C, for the melting curve program, every step, 5 s, and a constant increase by0.5°C. The amplification specificity was monitored by melting a curve after reaction (Supplementary Figure 1). All primers used in this research are listed in Supplementary Table 1, and *HvActin*gene was used as the internal standard (Kuang et al., 2019). Three biological replicates were measured for each stage, with three technical replicates conducted for each biological replicate to reduce the experimental error. The relative gene expression was calculated using 2^–ΔΔ^ ^CT^ method by taking the first stage S1 as the control (Livak and Schmittgen, 2001).

### Absolute Expression Analysis of *HvTIP1;1, HvTIP1;2, HvTIP2;3*, and *HvPIP2;1* in Multiple Plant Species

The coding sequences (CDS) of *HvTIP1;1, HvTIP1;2, HvTIP2;3*, and *HvPIP2;1* were used as a query to find the homologs in other five species (*Gossypium hirsutum, Oryza sativa, Zea mays, Nicotiana tabacum*, and *Solanum lycopersicum*). The absolute expression of the above four *AQP* genes in six plant species was analyzed according to Whelan et al. (2003) with some modifications. In brief, the primers for each *AQP* gene were specifically designed based on the highly conserved regions according to the sequence alignment of six species (Supplementary Table 2). Subsequently, the target fragments for the AQPs isoforms were amplified using these specific primers by PCR through KOD-Plus-Ver.2 (TOYOBO, Code: KOD-211), and the corresponding PCR products were tested by gel electrophoresis and melting curve analysis to confirm the specificity of the primers for each AQP isoform. In total, 24 specific fragments were finally obtained and then introduced into the PMD19-T vector. After the Sanger sequencing, the correct single clone of each AQP isoform was selected for the following absolute expression analysis. To form standard curves for absolute quantification, a series of dilutions (from 1 × 10^–1^ to 10^–5^ ng) of the plasmids were made and then detected by real-time PCR. The absolute RNA copy numbers were calculated from the standard curves using the Ct values of each sample. The expression levels of *TIP1;1, TIP1;2, TIP2;3*, and *PIP2;1* in the lodicules and petals of six species were quantified by absolute quantitative real-time PCR using the respective primers. Three biological replicates were measured for each flowering stage of either plant species.

### Evolutionary Bioinformatics

Forty-two species from algae to the land plants were selected to perform the comparative genetic similarity analysis. The genome sequences of these species were acquired from the public databases, including the National Center for Biotechnology Information (NCBI^13^), Ensemble plants (see text footnote 2), Congenie.org,^14^ and phytozome.^15^ Taking the *Arabidopsis* as the reference, the amino acid sequences of the whole genome from 42 species were aligned by the software BLASTP. According to the alignment results, candidate protein sequences were selected, which satisfied the criteria of E value < 10^–10^ and query coverage > 50%. The accession numbers of each sequence in the present study are listed in Supplementary Dataset 2. The sequence similarity heat map was generated by using TBtools (Chen C. et al., 2020).

To conduct the phylogenetic analysis of *AQP* genes across the entire plant kingdom using 1,231 species, the protein sequences of four *AQP* genes from the *Hordeum vulgare* L. (*TIP1;1, TIP1;2, TIP2;3*, and *PIP2;1*) were employed as the query sequences and matched to the sequences with the highest similarity in each species by BLASTP within OneKP database.^16^ The obtained sequences were then filtered with the criteria of E-value < 10^–10^ and query coverage > 50% (Deng et al., 2021). In addition, the duplicate values were also manually removed in Excel2019. Consequently, the remained∼900 protein sequences from OneKP database were used to conduct the evolutionary analysis. Multiple protein sequence alignment and phylogenetic analysis were performed by MAFFT^17^ and RAXML^18^ using the method of RAxML-VI-HPC (randomized axelerated maximum likelihood for high-performance computing), respectively. The phylogenetic analysis was annotated using the Interactive Tree of Life resource.^19^ The bootstrap values of phylogenetic trees were attached in the Supplementary Dataset 3. Protein structure and transmembrane domains were predicted by the SMART website (see text footnote 5). The protein 3D structure was depicted by SWISS-MODEL.^20^

## Results

### Identification of the AQP Gene Family in Barley and Evolutionary Similarity Analysis of It in Land Plants and Algal Species

To investigate the potential functions of AQPs in glume opening and closing in barley, the members belonging to HvAQPs across barley genome were identified in the present study. A total of 38 putative HvAQPs with 204∼367 amino acids were identified, 25 of which have been already presented in the previous study (Besse et al., 2011) (Supplementary Table 3). The phylogenetic analysis of these barley AQPs with those from *Arabidopsis* and rice revealed that barley AQPs could be clearly categorized into four clades, representing PIPs, TIPs, NIPs, and SIPs (Supplementary Figure 2A), including 16, 9, 11, and 2 members, respectively (Supplementary Figure 2B). Furthermore, it was observed that the motif compositions of HvAQPs were similar within the subfamily but differed dramatically between subfamilies (Supplementary Figure 2C). For instance, the motif 1 (NPA motif) was highly conserved in the subfamilies of HvPIPs, HvTIPs, and HvNIPs, whereas HvSIPs contained NPL or NPT motif instead of an NPA motif. Furthermore, the motifs 2, 3, 6, and 9 specifically existed in HvPIPs; the motif 5 only existed in the HvTIPs and HvNIPs; and the motif 8 was only present in HvPIP1. In addition, most members from HvPIPs, HvTIPs, and HvSIPs consisted of 3 exons, whereas HvNIPs consisted of 4-5 exons (Supplementary Figure 2D). These results indicated that the gene structures of HvAQPs are globally conserved but differed between subfamilies, in accordance with the phylogenetic results (Supplementary Figure 2A). Collinearity analysis of 38 *HvAQP*s on chromosomes showed that all examined *HvAQP* genes were randomly localized to seven chromosomes of barley (Supplementary Figure 3), and the *HvAQP* genes on 2H showed great collinear relationship with those on 5H and 6H, such as *HvPIP2;5* with *HvPIP1;2*, *HvPIP2;8*, *HvPIP2;9*, and *HvPIP2;10*. These results suggested that replication events may promote the gene expansion and further pry an essential driving force for the evolution of *HvAQP*s.

**FIGURE 1:**
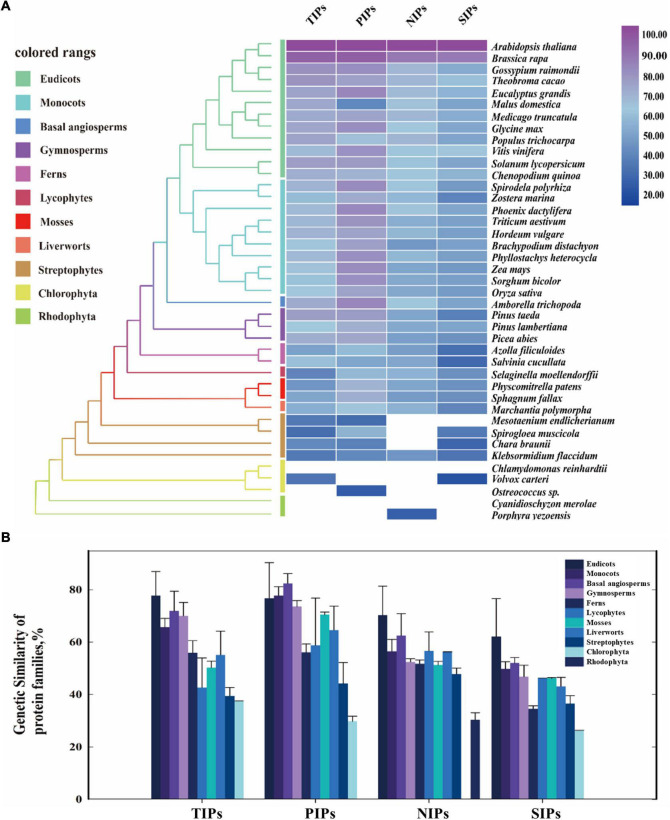
Evolutionary similarity of AQP protein families of the major clades in land plants and algal species. **(A)** The protein similarity heat map for the evolution of AQP families. A simplified tree was shown and color coded to the main clades of land plants and algal species. Colored rectangles indicate protein sequence similarity from 20% (blue) to 100% (purple). White squares indicate no proteins satisfied the selection criteria. **(B)** Comparative genetic similarity analysis was conducted with protein sequences of 42 plants and algal species that span from rhodophyta to eudicots. Colored rectangles indicate different plant groups. Candidate protein sequences were selected using BLASTP (NCBI) searches, and the results were filtered with an E-value threshold of 10^–5^ and sequence coverage of 50%.

The protein similarity of TIPs, PIPs, NIPs, and SIPs subfamilies across the major clades of plants ranging from Rhodophyta to eudicots was subsequently investigated by a comparative genetic analysis. To conduct this analysis, the genome databases of 42 major species of land plants and algae were employed (Figure 1A). It was shown that PIPs had the most conserved sequence characteristic among the four subfamilies, followed by TIPs and NIPs. Furthermore, TIPs, PIPs, and SIPs were traced to Chlorophyta, and present across Streptophyte algae to eudicots, although SIPs were not found in Streptophyte *Mesotaeniumendlicherianum*. On the other hand, large absence of NIPs in algae species was observed, although the homologs could be detected in Rhodophyte *Porphyrayezoensis* and Streptophyte *Klebsormidiumflaccidum* (Figure 1A). Moreover, AQP homologs identified in the examined Streptophytes displayed the highest similarity with those from land plants than the putative AQPs from Chlorophyta and Rhodophyta (Figure 1B). Taken together, although the origination of the four subfamilies of AQPs has been traced to different algae lineages, they seem to be conserved in the entire Plantae kingdom, indicating evolutionarily conserved functions in land plants.

### Transcriptomic Analysis of Differentially Expressed Genes in Lodicules During the Process of Glume Opening and Closing in Barley

**FIGURE 2:**
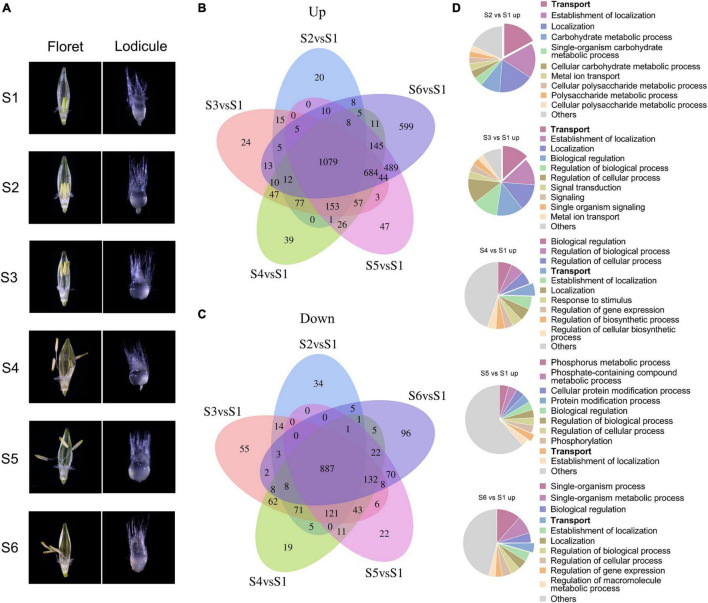
Transcriptomic analysis of differentially expressed genes during the glume opening and closing process. **(A)** Morphology of floret (left panel) and lodicule (right panel) at six stages (S1–S6) of flowering in barley. S1: the prior stage to glume opening with the green immature anthers and the small thin lodicules; S2: the initial stage of glume opening with the yellow mature anthers and the large thick lodicules; S3: the median stage of glume opening with half-elongated filaments (in the median space of the glumes) and half-enlarged lodicules; S4: the maximum stage of glume opening with fully elongated filaments (out of the glumes) and fully enlarged lodicules; S5: the median stage of glume closing with dehiscent anthers and dehydrating lodicules; and S6: the final stage of glume closing with pale anthers and fully dehydrated lodicules. Venn diagrams of upregulated **(B)** and downregulated **(C)** DEGs with | log_2_*^fold change^*| ≥ 1 and FDR < 0.05. **(D)** Gene Ontology analysis of upregulated DEGs was considered statistically significantly enriched when FDR < 0.05. All Go terms are listed in Supplementary Dataset 1.

Lodicules play critical roles during the anthesis process in gramineous plants such as barley (Tang et al., 2020), so it is necessary to elucidate the fundamental molecular mechanisms of lodicule swelling and withering during the process of glume opening and closing. According to our previous studies, the whole flowering process of barley could be divided into six typical stages: S1, the prior stage to glume opening with the green immature anthers and the small thin lodicules; S2, the initial stage of glume opening with the yellow mature anthers and the large thick lodicules; S3, the median stage of glume opening with half-elongated filaments (in the median space of the glumes) and half-enlarged lodicules; S4, the maximum stage of glume opening with fully elongated filaments (out of the glumes) and fully enlarged lodicules; S5, the median stage of glume closing with dehiscent anthers and dehydrating lodicules; and S6, the final stage of glume closing with pale anthers and fully dehydrated lodicules (Figure 2A). Therefore, the transcriptomic profile of lodicules was analyzed at these six stages using RNA sequencing in the present study.

Taking the S1 stage as the control, the Venn diagram and functional enrichment analysis of upregulated and downregulated differentially expressed genes (DEGs, | log_2_fold change| ≥ 1 and FDR ≤ 0.05) were analyzed at S2–S6 stages (Figure 2B). In total, there were 3,636 upregulated DEGs and 1,711 downregulated DEGs at S2–S6 stages, respectively. Among them, 1,079 upregulated DEGs and 887 downregulated DEGs were shared in all stages from S2 to S6. Furthermore, the number of upregulated DEGs showed an increasing tendency from 20 to 599 with the swelling and withering of lodicules during the process of glume opening and closing, whereas no such trend was observed for the downregulated DEGs (Figures 2B,C). Gene Ontology analysis (FDR < 0.05) revealed that the terms “transport,” “carbohydrate metabolic process,” “signal transduction,” and “localization” were mainly enriched in upregulated genes (Figure 2D), and “cellular process,” “macromolecule metabolic process,” “organic substance metabolic process,” and “cellular metabolic process” were obviously enriched in downregulated genes (Supplementary Figure 4). In addition, the upregulated DEGs involved in the “transport” process accounted for a substantial proportion of 3.5%–17% during the glume opening (Figure 2D). More importantly, there were 9–12 (6%–8%) AQPs in the “transport” term enriched at S2–S6 stages, respectively, suggesting the essential role of AQPs in the flowering process of barley.

**FIGURE 3:**
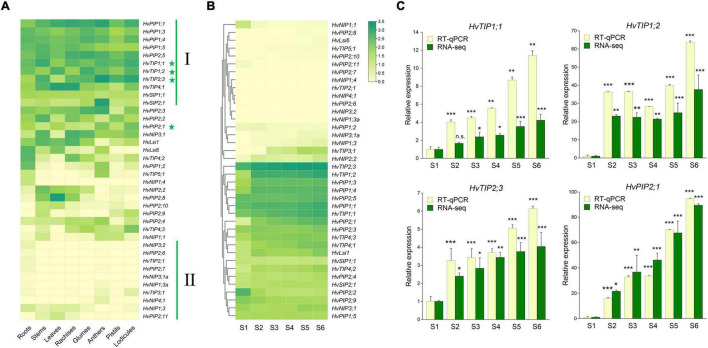
Expression patterns of *HvAQP*s in different tissues and stages by RNA-seq and real-time quantitative PCR (RT-qPCR). **(A)** The expression levels of *HvAQP*s (Based on TPM values) in roots, stems, leaves, rachises, glumes, anthers, pistils, and lodicules. **(B)** The expression patterns of *HvAQP*s at six stages (S1–S6) during the glume opening and closing process. **(C)** The relative expression ratio of four representative *HvAQP* genes in S2-S6 stages with the expression in S1 as the control. Data are shown as means ± SD (*n* = 3). * *p* < 0.01, ** *p* < 0.001, and *** *p* < 0.0001 using Dunnett’s test.

To evaluate the potential physiological roles of *HvAQP* genes during the anthesis in barley, the transcriptional levels of *HvAQP*s during the glume opening stage of barley were investigated by RNA sequencing analysis in eight tissues, including roots, stems, leaves, rachises, glumes, anthers, pistils, and lodicules. The results showed that most *PIP* and *TIP* subfamily genes showed much higher expression levels than *NIP* and *SIP* subfamily genes in barley (Figure 3A). There were 11 *HvAQP* genes (cluster I) expressed in all examined eight tissues. And 10 *HvAQP* genes (cluster II) displayed almost zero expression in all tissues with Transcripts Per Million (TPM) < 10. Among them, there were six *HvAQP*s, namely *HvTIP4;*1, *HvTIP4;2*, *HvPIP2;2*, *HvPIP2;8*, *HvLsi1*, and *HvLsi6* showed significantly higher expression levels in the vegetative tissues than those in the reproductive organs (Figure 3A and Supplementary Figure 5A), indicating their importance for the vegetative growth and development. Moreover, *HvSIP2;1* and *HvTIP5;1* exhibited superior expression in anthers, suggesting their potential roles in the development and fertility of anthers. Furthermore, the transcripts of *HvTIP1;1* were mostly abundant in stems and lodicules, and *HvTIP1;2* was mainly expressed in glumes, leaves, and lodicules. *HvTIP2;3* was highly expressed in anther and lodicules. Besides, *HvPIP2;1* and *HvTIP4;3* showed the highest expression in lodicules, which was about 1.5–23. and 3.2–137.1 times of the expression in other tissues, respectively (Figure 3A). All of these *TIP* or *PIP* genes exhibited a relatively high expression level in the lodicules, indicating great potentiality of these genes in affecting the glume opening by controlling the water transport in lodicules.

Accordingly, the expression profile of the above 38 *Hv*AQP genes during the glume opening and closing in barley was further examined (Figure 3B). There were ten differentially expressed *AQP* genes were identified during flowering in barley (Supplementary Table 4). Among the upregulated genes, *HvTIP1;1, HvTIP1;2, HvTIP2;3*, and *HvPIP2;1* not only exhibited relatively higher expression levels in lodicules (Figure 3A and Supplementary Figure 5B) but also showed an elevated expression tendency during the six stages of flowering (Figures 3B,C and Supplementary Figure 5B). The dynamic expression profiles were subsequently confirmed by RT-qPCR during the flowering (Figure 3C). These results suggested that *HvTIP1;1, HvTIP1;2, HvTIP2;3*, and *HvPIP2;1* could be the crucial candidate *HvAQP* genes involved in the water transport in lodicules during the glume opening and closing in barley.

Therefore, two intriguing questions arose in the present study: Are these AQPs functionally conserved during the flowering process in plants? If yes, is the conserved function of AQPs in plant flowering linked with their evolutionary conservation?

### The Expression Tendency of *TIP1;1, TIP1;2, TIP2;3*, and *PIP2;1* During the Flowering in Angiosperms

**FIGURE 4:**
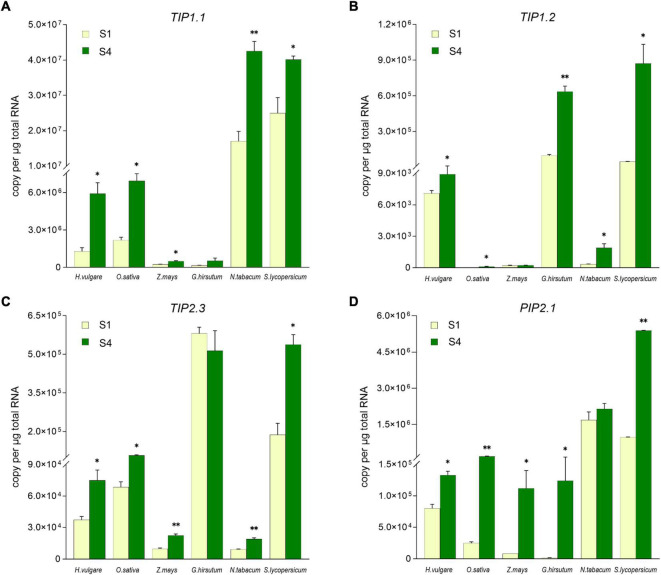
Comparison of the *TIP1;1*, *TIP1;2*, *TIP2;3*, and *PIP2;1* expression level in lodicules or petals in six species by absolute quantitative real-time PCR. The expression level of *TIP1;1* **(A)**, *TIP1;2* **(B)**, *TIP2;3* **(C)**, and *PIP2;1* **(D)** in lodicules or petals was compared in *Gossypium hirsutum, Oryza sativa*, *Zea mays*, *Nicotiana tabacum*, *Solanum lycopersicum*, and *Hordeum vulgare*. Data are means ± SD (*n* = 3). * *p* < 0.05 and ^**^ *p* < 0.01 using Student’s *t*-test.

To explore whether *TIP1;1*, *TIP1;2*, *TIP2;3*, and *PIP2;1* play the conserved role in plant flowering, the transcriptional levels of their putative homologs during the flowering process were compared in 6 angiosperm species, including 3 monocots (*Hordeum vulgare*, *Oryza sativa*, and *Zea mays*) and 3 eudicots (*Gossypium hirsutum*, *Nicotiana tabacum*, and *Solanum lycopersicum*) (Supplementary Figure 6). According to the previous molecular genetic studies, it has been considered that lodicules are the grass-specific floral organs analogous to eudicot petals (Yoshida, 2012). Thus, lodicules from the 3 monocots and petals of the 3 eudicots at the prior stage (S1) and the maximum stage (S4) of flowering were harvested for the expression levels determination. As shown in Figure 4, the absolute expression levels of the *AQP* candidates varied among the examined plant species. In detail, the transcripts of *TIP1.1* were much fewer in maize and cotton, while the transcripts reached to 10^7^ copies per ug total RNA in the petals of tobacco and tomato; the accumulation of *TIP1.2* transcripts in rice, maize, and tobacco was extremely low but high in barley, cotton, and tomato. The expression of *TIP2.3* was relatively lower in maize and tobacco but higher in the other 2 eudicots. Almost all *AQP* candidates were significantly higher at the S4 stage than those at the S1 stage, except *TIP1.1* and *TIP2.3* in cotton, *TIP1.2* in maize, and *PIP2.1* in tobacco. The abundance of *PIP2.1* in all examined species examined was high at the S4 stage (Figure 4). Furthermore, the expression of four candidate *AQP* genes was all significantly responding to the process of flowering in six investigated plant species. For instance, almost all of the *AQP* genes in lodicules were significantly upregulated during glume opening in the examined monocots with an exception of *TIP1;2* in maize, being consistent with the results of RNA-seq analysis in barley (Figure 3B). Likewise, the expression levels of 4 *AQP* genes in petals at Stage S4 were dramatically higher than those at Stage S1 in the 3 eudicot species, except *TIP1;1* and *TIP2;3* in cotton and *PIP2;1* in tobacco (Figure 4). In addition, we also found that the amount of background copy number of *TIP1;1* was at least ten times higher than *TIP1;2*, *TIP2;3*, and *PIP2;1* in the investigated plant species (Figure 4). Taken together, the candidate *AQP* genes especially *TIP1;1*, *TIP1;2, TIP2;3*, and *PIP2;1* share a very similar expression pattern during the flowering process in both monocots and eudicots, suggesting a functional conservation of them in plant flowering.

### Molecular Evolution of *TIP1;1, TIP1;2, TIP2;3, and PIP2;1* for Flowering

**FIGURE 5:**
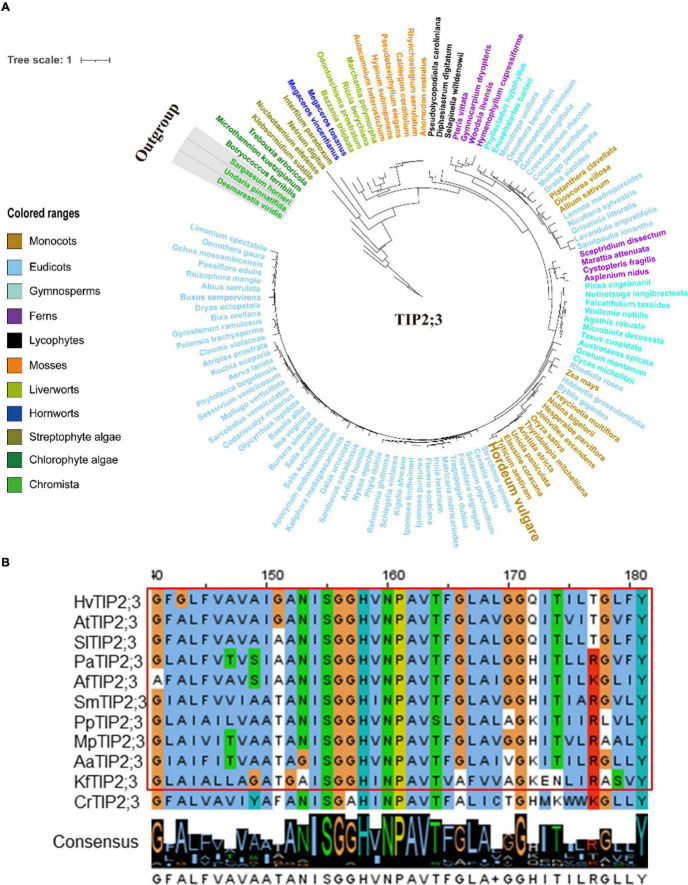
Molecular evolutionary analysis of the aquaporins TIP2;3 in plants and algae. **(A)** The phylogenetic tree of TIP2;3 proteins in representative species of major lineage of plants and algae (See Supplementary Figure 9A for all OneKP species). The maximum likelihood (ML) method was used to construct the tree. Clades are indicated by different colors. **(B)** Conserved motif alignment of TIP2;3 in eleven representative plants and algal species. *Hv, Hordeum vulgare; At, Arabidopsis thaliana; Sl, Solanum lycopersicum; Pa, Piceaabies; Af, Azolla filiculoides; Sm, Selaginella moellendorffii; Pp, physcomitrella patens; Mp, Marchantia polymorpha; Aa, Anthoceros angustus; Kf, Klebsormidium flaccidum; Cr, Chlamydomonas reinhardtii*.

**FIGURE 6:**
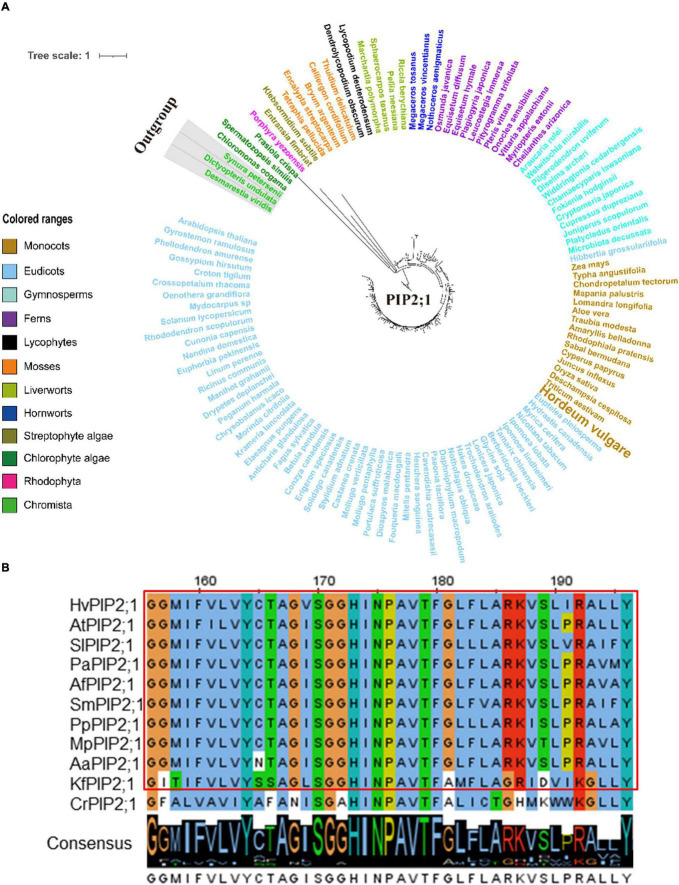
Molecular evolutionary analysis of the aquaporins PIP2;1 in plants and algae. **(A)** The phylogenetic tree of PIP2;1 proteins in representative species of major lineage of plants and algae (See Supplementary Figure 10A for all OneKP species). The maximum likelihood (ML) method was used to construct the tree. Clades are indicated by different colors. **(B)** Conserved motif alignment of PIP2;1 in eleven representative plants and algal species. *Hv, Hordeum vulgare; At, Arabidopsis thaliana; Sl, Solanum lycopersicum; Pa, Piceaabies; Af, Azolla filiculoides; Sm, Selaginella moellendorffii; Pp, physcomitrella patens; Mp, Marchantia polymorpha; Aa, Anthoceros angustus; Kf, Klebsormidium flaccidum; Cr, Chlamydomonas reinhardtii*.

Since TIP1;1, TIP1;2, TIP2;3, and PIP2;1 displayed a conserved function in regulating the flowering process in plants, one might ask that would such functional conservation be associated with the evolutionary conservation for these AQP proteins? In order to further understand the evolutionary process of TIP1;1, TIP1;2, TIP2;3, and PIP2;1, phylogenetic analysis of these proteins was performed across the entire plant kingdom using the OneKP database (Leebens-Mack et al., 2019). As shown in the phylogenetic tree generated by using the transcriptomes of 1,231 species, four AQPs were detected in 75–78% of plants and algae (Supplementary Figures 7A–10A and Supplementary Table 5), and they were all traced to Chlorophyta, indicating an early evolution of them in plants (Figures 5, 6 and Supplementary Figures 7–12). Among them, TIP1;1 and TIP1;2 experienced a very similar evolutionary process that both of them were basal to *Microthamnion Kuetzigianum*, and the angiosperm cluster was grouped into two subclusters, consisting of specific monocots and eudicots (Supplementary Figures 11A, 12A). These results indicated that both TIP1;1 and TIP1;2 have the same ancient origin in lower plants but have recently diverged into two evolutionary branches in angiosperms. Unlike the above TIPs, the origin of TIP2;3 could be traced back to Chlorophyta species *Botryococcusterribilis* and then experienced a monophyletic evolutionary lineage to lycophytes (Figure 5A and Supplementary Figure 9A). Thereafter, TIP2;3 in the vascular plants was diverged into two branches, containing their specific ferns, gymnosperms, monocots, and eudicots. It could be suggested that TIP2;3 in the vascular plants descended from a polyphyletic evolutionary lineage since ferns, which could be dated back to approximately 200 million years ago before angiosperms (Cai et al., 2021). The evolutionary pathway of PIP2;1 candidate presented a higher sequence conservation in comparison with three TIPs in angiosperms (Figure 6A and Supplementary Figure 10A). Although all the four candidate AQPs in the present study showed the visible sequence variation between algae and bryophytes (such as liverworts and hornworts), a more dramatic change was observed for PIP2;1 (45.6% to 78.8%) (Supplementary Figure 13). Furthermore, PIP2;1 homologs in seed plants were all basal to ferns, indicating a recent origin of PIP2;1 in seed plants from ferns other than lycophytes. In angiosperms, surprisingly, PIP2;1 orthologs presented a highly sequence conservation in comparison to the 3 TIPs (Figure 6A and Supplementary Figure 10A), being consistent with the previous protein similarity analysis (Figure 1).

The protein similarity and 3D structure of the 4 *HvAQP*s highly expressed in barley lodicules were calculated by the SWISS-MODEL tool using the AtTIP2;1 (5i32.1.A) and SoPIP2;1 (4jc6.2.D) as the template, respectively (Waterhouse et al., 2018). The core functional domains (MIPs) of the 4 candidates displayed extremely high similarity among the typical plant species from Streptophyte algae to eudicots, which were consistent with the predicted 3D structures (Supplementary Figures 7B,C–10B,C). Constantly, more variations of amino acids were observed from chlorophyte algae (*Chlamydomonas reinhardtii*) than those from Streptophyte algae compared with the putative AQPs from land plants (Figures 5B, 6B and Supplementary Figures 11B, 12B).

In addition, an increment of the numbers of AQP members from algae to terrestrial plants was observed. For instance, the members of 3 TIP subfamily AQPs in Streptophyte algae and hornworts showed a statistically significant increasing trend from approximately 30 to 73%, and PIP2;1 increased from 19 to 67% (Supplementary Table 5), indicating the crucial roles of AQPs in the process of terrestrial adaptation.

Taken together, the putative homologs of 4 candidate AQPs (TIP1;1, TIP1;2, TIP2;3, and PIP2;1) experienced the similar evolutionary process from algae to angiosperms, and their protein similarity in land plants is largely conserved during evolution, especially in the angiosperm species, in line with their conserved function in controlling the flowering process.

## Discussion

### AQP Subfamilies Experience a Similar Evolutionary Process Across the Plant Kingdom, and They Are Highly Conserved in Land Plants

Green plants (Viridiplantae) consist of estimated 450,000–500,000 species on the earth, including green algae and embryophytic land plants. They encompass a high level of ecological diversity and evolutionary timescales, which play important roles in all terrestrial and most aquatic ecosystems (Corlett, 2016; Kumar et al., 2017). Phylogenomics, which uses genomic data to produce phylogenetic relationships among organisms, has been widely used to resolve species relationships as well as the evolution of genomes, gene families, and gene function (Eisen, 1998; Philippe et al., 2005). Recently, a robust phylogenomic framework of green plants has been established with 1,231 species by using the vegetative transcriptomes and the published genomes, enabling a new and effective data pool (OneKP^21^) for examining the evolution of green plants (Leebens-Mack et al., 2019).

AQPs, which facilitate the passive movement of water and small solutes across biological membranes, are regarded an ancient family of water channel proteins present in all kingdoms of life, suggesting their essential role in basal life functions (Bezerra-Neto et al., 2019). Despite the phylogenetic relationship of AQPs has been extensively studied in plants (Finn and Cerdà, 2015), the systematic research on the evolution of AQPs using a broad of species from algae to land plants is very rare. In the present study, the phylogenetic analysis of AQPs was performed across the entire plant kingdom using the OneKP data pool, together with 3 published genomes for the first time. The comparative genetic similarity analysis of AQP proteins across 42 plant species revealed that TIPs, PIPs, and SIPs could be traced to Chlorophyta and present across Streptophyte algae to eudicots, indicating an ancestral evolution of these AQP subfamilies (Deng et al., 2021). However, the NIPs subfamily was observed largely absent in the algae clade (Figure 1), which suggests that the NIPs subfamily is likely originated from the basal land plants, much later than the other AQP subfamilies, such as PIPs, TIPs, and SIPs (Anderberg et al., 2011). It is generally accepted that NIPs are acquired, diversified, and sub-functionalized during the evolutionary emergence of the embryophyta (Roberts and Routray, 2017). In view of the absence of aquaglyceroporins in the early land plants and glycerol transport activity of NIPs (Pommerrenig et al., 2020), the origin of NIPs likely derives from a single event of horizontal gene transfer from bacteria to plants (Zardoya et al., 2002).

Based on the narrow selectivity filter regions [the aromatic/Arg(ar/R) filter], the subfamilies PIP, TIP, NIP, and SIP can be further subdivided into the smaller subgroups (Wallace and Roberts, 2004). In barley, we have identified 38 AQPs, including 16 PIPs, 9 TIPs, 11 NIPs, and 2 SIPs (Supplementary Figure 2). As same as in the other plant species like *Arabidopsis* and rice, the PIP subfamily in barley is subdivided into two subgroups, PIP1 and PIP2, generally representing the highest abundance of AQPs in plants (Supplementary Figure 2A). Such divergence could be traced back prior to the emergence of land plants (Groszmann et al., 2017). In addition, the phylogenetic tree of PIP2;1 conducted with the OneKP database revealed that PIP2;1 homologs presented a highly sequence conservation in comparison with those of three TIPs (Figure 6A and Supplementary Figure 10A), being consistent with the protein similarity analysis (Figure 1). Such high conservation of PIPs in plants indicates that they are evolved earlier in the evolutionary history of crop plants and of great importance in plant physiology (Hussain et al., 2020). Members of the TIP subfamily were clearly distinguished into five subgroups (TIP1-TIP5) based on the phylogenetic tree of AQP proteins in barley, rice, and *Arabidopsis* (Supplementary Figure 2A). In these species, TIP2, TIP3, and TIP4 represent the basal subgroups, with TIP1 and TIP5 as sister subgroups to TIP3 and TIP2, which is consistent with the previous study (Abascal et al., 2014; Laur and Hacke, 2014). It has been reported that each of TIP subgroups shows the specific gene duplication from the internal family, and such major expansion likely originates in the ancestor of seed plants, such as Lycopodiophyta and Bryophyta, after divergence from the ferns (Danielson and Johanson, 2008; Anderberg et al., 2012; Groszmann et al., 2017). Furthermore, the number of species containing the above four AQPs was increased by 2–3 times from Streptophyte algae to hornworts and maintained over 90% in seed plants (Supplementary Table 5), suggesting the substantial proliferation of these AQPs in the land plant species (Groszmann et al., 2017), as they are highly water permeable (Prado and Maurel, 2013) and play the important roles in different physiological processes involved in the transport of water to or from roots, leaves, and reproductive organs (Bezerra-Neto et al., 2019). Moreover, the visible sequence variation in TIP and PIP subfamily AQPs between algae and bryophytes (such as liverworts and hornworts) was also observed (Supplementary Figure 13). It can be expected that these PIPs and TIPs have been developed by the ancestors of land plants for adaptation to the variable and complex terrestrial environments (Li et al., 2015). Furthermore, we found that the putative homologs of PIP and TIP showed the high similarity in the regions of core functional domains and protein 3D structure (Supplementary Figures 7B,C–10B,C). Similar results were obtained by the comparative genetic similarity analysis on PIP and TIP subfamilies using 42 typical plant lineages, ranging from Rhodophyta to eudicots (Figure 1). Taken together, the AQP members from PIP and TIP subfamilies are relatively evolutionarily conserved in land plants, indicating that they may play an essential role in the long-term natural selection of plants (Li et al., 2019) and have a high level of conservation in their biofunctions in plants, such as petal development and movement in eudicots (Azad et al., 2004), lodicules swelling in monocots (Figures 3, 4), anther dehiscence (Bots et al., 2005), internode elongation (Muto et al., 2011), and lateral root emergence (Reinhardt et al., 2016).

### AQPs Play the Critical Role in the Glume Opening and Closing of Gramineous Plants as Well as in the Petal Expansion of Eudicots

Glume opening is the key process for the sexual reproduction in gramineous plants. The physiological basis of glume opening is, actually, the rapid and uniform enlargement of the lodicules, which is attributed to the rapid influx of water by the increased internal osmotic pressure (Liu et al., 2017). It is reported that the altered osmotic pressure is the main driving force for the swelling of lodicules, which is thought to be related to the metabolism and flux of carbohydrates (mainly including total soluble sugars), amino acids, calcium, and potassium ions (Beauzamy et al., 2014; Chen J. et al., 2020). This is consistent with our significant enriched biological processes of DEGs in lodicules during floret opening, mainly including “transport,” “cellular carbohydrate metabolic process,” and “signal transduction” (Figure 2D). With a rapid rise of the osmotic pressure, the drastic influx of water occurs in the lodicule cells (Chen J. et al., 2020). The swollen lodicules then push the palea and the lemma, respectively, which leads to the glume opening (Honda et al., 2005; Yadav et al., 2007). Thus, it is of great significance to elucidate the regulatory mechanisms underlying the water balance in lodicules during the flowering process in gramineous plants. It has been established that rapid water flow and uptake across biological membranes are mainly controlled by AQPs (Kong et al., 2018), which participate in the regulation of diverse developmental processes in plants, including flowering (Maurel et al., 2015), cell elongation, and stomatal movement (Johansson et al., 2000). In recent decades, with the availability of the whole genome sequencing data, members of the AQP family have been identified in many plant species (Li H. et al., 2014), such as maize (Bari et al., 2018), wheat (Pandey et al., 2013), melon (Lopez-Zaplana et al., 2020), and tomato (Reuscher et al., 2013). However, very few studies have focused on the identification, characterization, and functional analysis of barley AQPs. In the present study, we have identified a total of 38 AQP family members across the barley genome and evaluated their function in glume opening and closing by profiling the gene expression of *AQP*s in lodicules during the flowering process in barley *via* RNA-sequencing, RT-qPCR, and the absolute RNA expression assay.

The gene expression profile of 38 *HvAQP*s in eight tissues at the anthesis stage revealed that the transcripts of *PIP* and *TIP* subfamily members in barley were highly abundant in all examined tissues (Figure 3A), which is consistent with the observations in other species, such as rice (Nguyen et al., 2013). Given that PIPs and TIPs are highly permeable to water (Danielson and Johanson, 2010), their high transcript abundance implied their crucial roles in intracellular, intercellular, organ, and whole-plant water balance in barley. For instance, *HvPIP1;2* and *HvTIP4;2* showed advantage expression in roots, indicating they might play key roles in water absorption and transport in root cells, which has been extensively reported in the other plant species like *Arabidopsis* (Postaire et al., 2010) and *Eutremasalsugineum* (Qin et al., 2019). Furthermore, *HvTIP1;1*, *HvTIP1;2*, *HvTIP2;3*, and *HvPIP2;1* exhibited high expression levels both in lodicules and anther, indicating their important roles during the flowering process. Likewise in maize, it was reported that *ZmTIP1* expression was highest in the lodicule primordia in the tassel spikelet (Chaumont et al., 1998). All these results suggest the functional differentiation of AQPs during plant adaption to the complex and changing environment.

Previous molecular genetic studies have revealed that lodicules are grass-specific floral organs analogous to eudicot petals (Yoshida, 2012). In eudicots, petal expansion is mainly driven by cell expansion during flower opening (Guterman et al., 2002), which is a process of the rapid water uptake of the petal to further push the sepals separately for blooming (Yoshida and Nagato, 2011). There have been many studies reported the involvement of AQPs in flowering of eudicots. In tulips, the opening and the closing of flowers were related to the water absorption of the petal tissues, which may be involved in the activity of AQPs (Azad et al., 2004). In *Arabidopsis*, various members of the TIP subfamily, such as *AtTIP1;3*, *AtTIP2;1*, *AtTIP3;1*, and *AtTIP5;1* were specifically expressed in petals, indicating their importance in flowering (Alexandersson et al., 2005). Likewise, *RhTIP1;1* was predominantly expressed in the petals of rose flowers (Xue et al., 2009), whereas *LoPIP1* and *LoPIP2* were strongly expressed in young petals of lily (Tong et al., 2013). Moreover, it was also found that *RhPIP2;1* is involved in ethylene-regulated petal expansion (Ma et al., 2008). It can be expected that PIP and TIP subfamily AQPs probably play an essential role in flower opening through the regulation of petal expansion *via* water uptake. Therefore, the gene expression profiling of *AQPs* in lodicules would be greatly helpful for understanding the mechanical basis of glume opening and closing in gramineous crops. In the present study, both RNA-sequencing and RT-qPCR analysis revealed that *HvTIP1;1, HvTIP1;2, HvTIP2;3*, and *HvPIP2;1* exhibited a gradually elevated expression level during the six stages of flowering in barley (Figures 3B,C and Supplementary Table 4). In parallel to this, the lodicules experienced the process of swelling and withering (Figure 2A), indicating the essential role of AQPs in controlling the water flow both into and out the lodicules during the process of glume opening and closing. Furthermore, according to the absolute quantitative expression analysis in multiple species, it was also found that the putative homologs of these four *AQP* genes were mostly upregulated in responding to the floret opening in both monocots (barley, rice, and maize) and eudicots (cotton, tobacco, and tomato), suggesting a conserved function of them in the flowering process of angiosperms (Figure 4). In addition, the transcriptional level of *TIP1;1* was at least ten times higher than *TIP1;2*, *TIP2;3*, and *PIP2;1* (Figure 4). Therefore, it can be concluded that the AQP members from PIP and TIP subfamilies, especially TIP1;1, TIP1;2, TIP2;3, and PIP2;1, may be critical to flowering in both monocots and eudicots.

### The Potentiality of Using AQPs for Artificial Regulation of Glume Opening and Closing in Gramineous Crops to Optimize Hybrid Seed Production

Artificial regulation of glume opening and closing is an important attempt to obtain the flowering synchrony between parents in hybrid seed production. Indeed, a series of approaches, including adjusting the sowing date, foliar spraying urea and potassium, and application of plant growth regulators like methyl jasmonate, have been applied to regulate the flowering activities of parental lines in hybrid seed production of gramineous crops like rice (Mondo et al., 2016a; Gaballah et al., 2021). Unfortunately, very limited success has been achieved. The fundamental reason lies in the lack of systematic and in-depth pieces of research on the flowering process of gramineous crops. Therefore, fully understanding the mechanism of glume opening and closing in gramineous crops is of great significance for optimizing hybrid seed production. In the present study, we have identified four elite *AQP* genes, namely *HvTIP1;1, HvTIP1;2*, *HvTIP2;3*, and *HvPIP2;1*, which were not only highly expressed in lodicule tissues but also showed a gradually elevated expression level during the six stages of flowering in barley (Figure 3B and Supplementary Table 4). Furthermore, the putative homologs of these genes not only showed the large evolutionary conservation in land plants but also exhibited the functional conservation in view of upregulation tendency toward responding to the floret opening in the other monocots (rice and maize) and eudicots (cotton, tobacco, and tomato) (Figure 4). All these results suggested that *TIP1;1, TIP1;2*, *TIP1;2*, and *PIP2;1* could be the potential candidate *AQP* genes to regulate the flowering process by controlling the water transport in floral organs. It has been reported that overexpression of *Panax ginseng TIP1;1* in *Arabidopsis* led to general acceleration flowering when compared with the wild type (Peng et al., 2007). On the other hand, the silence of rose *Rh-PIP2;1* was found to result in the great inhibition in petal expansion, thereby leading to the abnormal flower opening (Ma et al., 2008). More recently, we have also found that *HvTIP1;1*, *HvTIP1;2*, *HvTIP2;3*, and *HvPIP2;1* were localized at tonoplast and plasma membrane, respectively, and exhibited high permeability to water when they were expressed in *Xenopus laevis* oocytes (unpublished data). Therefore, it is of great potentiality to utilize these four *AQP* genes to artificially regulate the glume opening and closing in gramineous crops like barley and rice. One approach to achieve this goal is to overexpress the candidate AQP genes in gramineous crops, which may accelerate the glume opening process and maintain the lodicules swelling for longer time to enhance the pollination rate of the ovary for optimal hybrid seed production. Recent research on the poplar *AQP* gene *PtoPIP1;1* has shown that the constitutive overexpression of it in *Arabidopsis* accelerated the flowering process of the transgenic plants (Leng et al., 2021). However, further studies are required to comprehensively investigate the characteristics, specific functions, and regulatory mechanisms of these four candidate AQPs prior to the utilization of them.

## Conclusion

In the present study, 38 HvAQP proteins, including 16 PIPs, 9 TIPs, 11 NIPs, and 2 SIPs, were identified within the whole barley genome. The comparative genetic similarity analysis of these four subfamilies across 42 typical plant lineages, ranging from Rhodophyta to eudicots, revealed that PIP and TIP subfamilies could be traced to Chlorophyta, and they were highly evolutionarily conserved in land plants. In barley, *AQP* genes, e.g., *HvTIP1;1*, *HvTIP1;2*, *HvTIP2;3*, and *HvPIP2;1*, were identified to be abundantly expressed in lodicules and significantly upregulated in response to the flowering process. Similar results were obtained for the putative homologs of these four *AQP* genes in the lodicules and petals of the other monocot and eudicot species, suggesting a high conservation of TIP1;1, TIP1;2, TIP2;3, and PIP2;1 in regulating flowering in both monocots and eudicots. Furthermore, the phylogenetic analysis using 1,231 plant species from OneKP database revealed that the homologs of the four candidate AQP proteins were highly conserved during the evolution, especially in the angiosperm species, in paralleling with their conserved function in controlling the flowering process. Taken together, it could be concluded that the highly evolutionary conservation of TIP1;1, TIP1;2, TIP2;3, and PIP2;1 plays important roles in flowering of land plants, providing potentiality to utilize them for artificially controlling the flowering process in plants. However, the characteristics, specific functions, and regulatory mechanisms of these four candidate AQPs are required to be further studied using biotechnological approaches.

## Data Availability Statement

The original contributions presented in the study are publicly available. This data can be found here: National Center for Biotechnology Information (NCBI) BioProject database under accession number PRJNA752285.

## Author Contributions

FZ, Z-HC, and FD conceived the study. QL performed the experimental work. QL, TT, WJ, and JC analyzed and visualized the data. FZ, XW, and YO supervised the experiments. QL and FZ drafted the manuscript. All authors read and revised the manuscript.

## Conflict of Interest

The authors declare that the research was conducted in the absence of any commercial or financial relationships that could be construed as a potential conflict of interest.

## Publisher’s Note

All claims expressed in this article are solely those of the authors and do not necessarily represent those of their affiliated organizations, or those of the publisher, the editors and the reviewers. Any product that may be evaluated in this article, or claim that may be made by its manufacturer, is not guaranteed or endorsed by the publisher.

## Footnotes

1
http://faostat.fao.org/

2
http://plants.ensembl.org/index.html

3
http://pfam.xfam.org/family/PF00230

4
https://www.ncbi.nlm.nih.gov/Structure/bwrpsb/bwrpsb.cgi

5
http://smart.embl-heidelberg.de/

6
http://www.cbs.dtu.dk/services/TMHMM/

7
http://web.expasy.org/protparam/

8
http://www.csbio.sjtu.edu.cn/bioinf/plant-multi/

9
https://www.uniprot.org/uniprot/

10
http://meme-suite.org/

11
https://www.omicstudio.cn/tool

12
http://systemsbiology.cau.edu.cn/agriGOv2/

13
https://www.ncbi.nlm.nih.gov/genome/

14
https://congenie.org/

15
https://phytozome.jgi.doe.gov/pz/portal.html

16
www.OneKP.com

17
https://mafft.cbrc.jp/alignment/server/

18
https://www.phylo.org/portal2/tools.action

19
http://itol.embl.de

20
https://swissmodel.expasy.org/

21
https://db.cngb.org/OneKP/
