# Supplementary material for: Highly Conserved Evolution of Aquaporin PIPs and TIPs Confers Their Crucial Contribution to Flowering Process in Plants
Source: Front Plant Sci. 2022 Jan 4;12:761713. doi: 10.3389/fpls.2021.761713 (PMC8764411; doi:10.3389/fpls.2021.761713)
Supplement: Supplementary file 2 [file Data_Sheet_2.pdf]

Supplementary Material

1 Supplementary Figures and Tables

1.1 Supplementary Figures

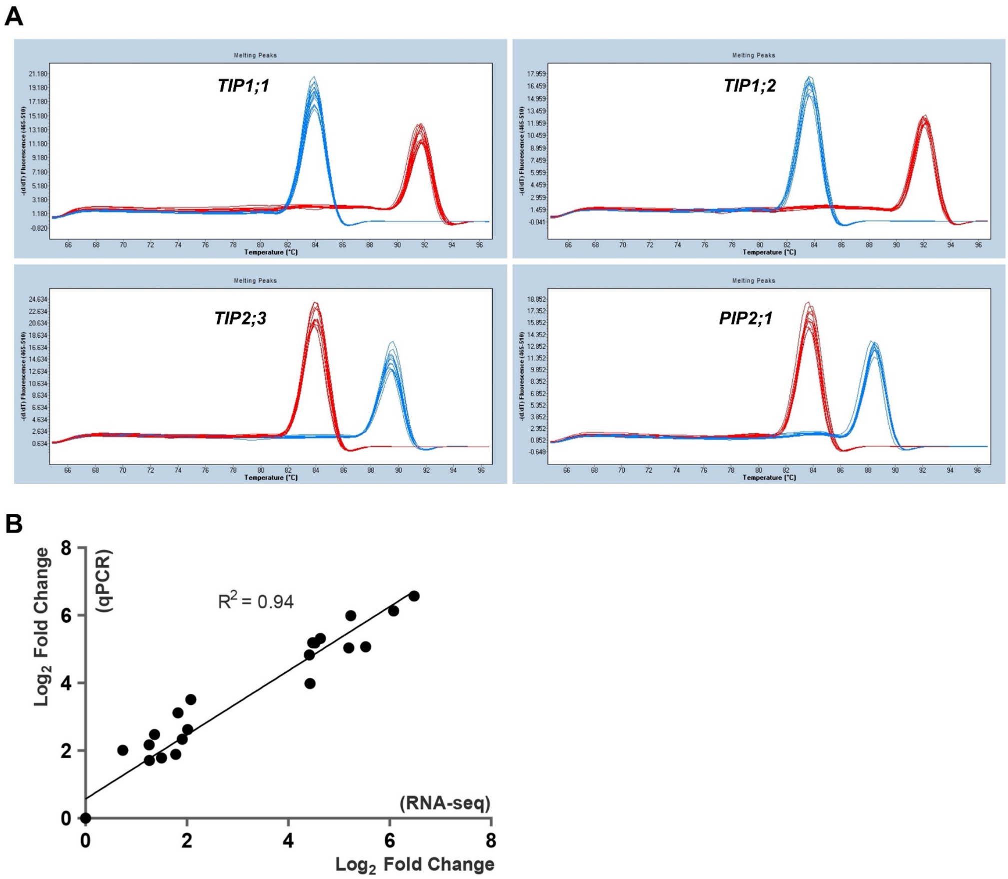

Supplementary Figure 1. The melting curve analysis and the concordance of RNA-seq and RT-qPCR data. (**A**) The melting curves of *HvTIP1;1*, *HvTIP1;2*, *HvTIP2;3* and *HvPIP2;1* primers for RT-qPCR analysis showed the high specificity. (**B**) Correlation of RNA-seq (x-axis) and RT-qPCR data (y-axis) using the log2 fold change measure of the genes differentially expressed P-value across the two gene-expression platforms under correlation analysis.

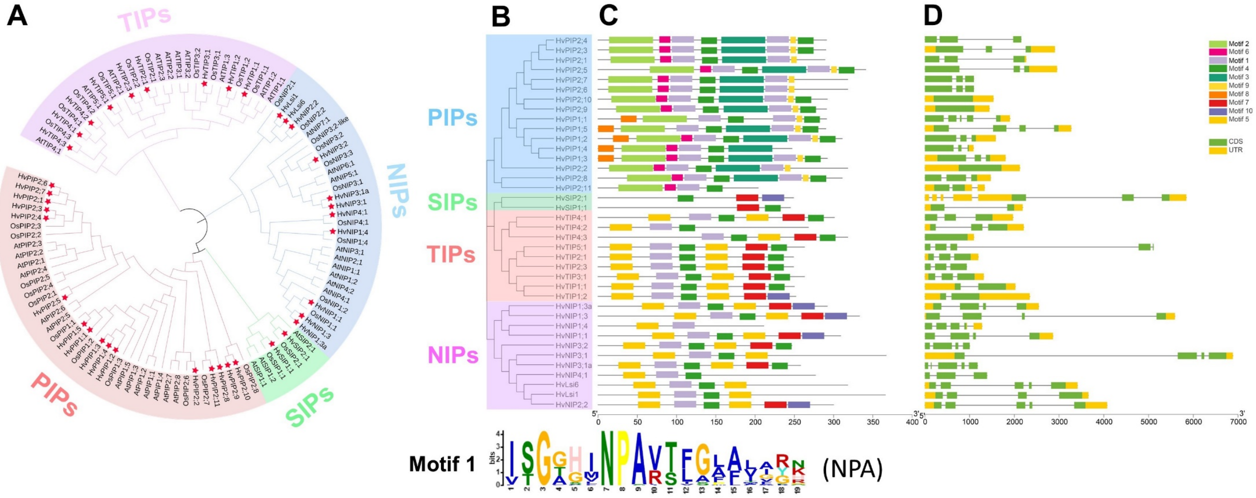

Supplementary Figure 2. Phylogenetic analysis and gene structures of *Hordeum vulgare* aquaporin (*HvAQP*) genes. (**A**) Phylogenetic analysis of aquaporin proteins in barley, *Arabidopsis* and rice. A Maximum Likelihood tree was constructed using ClustalW and MEGA7.0 software. 106 aquaporin proteins were classified as 40 plasma membrane intrinsic proteins (PIPs) in the pink tree, 29 tonoplast intrinsic proteins (TIPs) in the purple tree, 30 nodulin 26-like intrinsic proteins (NIPs) in the blue tree, and 7 small basic intrinsic proteins (SIPs) in the green tree. Red stars mark the barley members. *At* denotes *Arabidopsis*, *Os* for rice, and *Hv* for barley. (**B**) Phylogenetic tree of 38 HvAQP proteins. The unrooted Neighbor-joining phylogenetic tree was constructed using the full-length amino acid sequences of 38 HvAQP proteins. (**C**) Arrangements of conserved motifs in the HvAQP proteins. Ten predicted motifs are represented by different colored boxes, and motif sizes are indicated by the scale at bottom. The conserved motif was shown in the bottom of the picture. (**D**) Exon-intron organization of *HvAQP* genes. Green boxes represent exons and black lines represent introns. The untranslated regions (UTR) of *HvAQP* genes are indicated by yellow boxes. The sizes of exons and introns can be estimated by the scale at bottom.

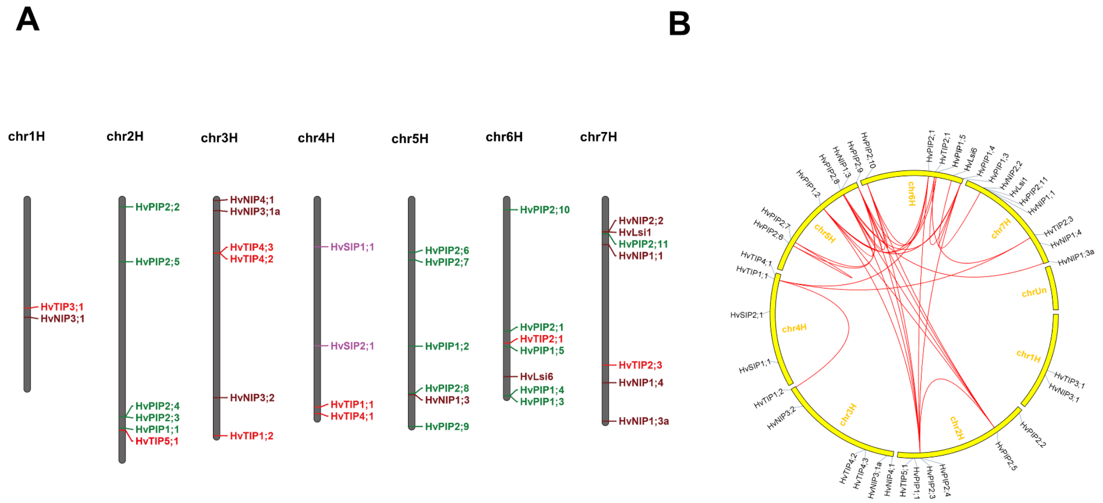

Supplementary Figure 3. The chromosomal location and syntenic analysis of *HvAQP* genes. (**A**) Distribution of *HvAQP*s on seven barley chromosomes. Physical position determined by *Hordeum vulgare* L. reference sequence. Chr1H-7H represent the chromosome 1H to 7H. The red, brown, green and purple colors in the right side indicate TIPs, NIPs, PIPs and SIPs, respectively. (**B**) Schematic representations for the chromosomal distribution and inter chromosomal relationships of barley *AQP* genes. The red lines indicate the putatively duplicated *AQP* gene pairs with the protein/coding sequence similarity of 75%. The chromosome number is indicated at the bottom of each chromosome.

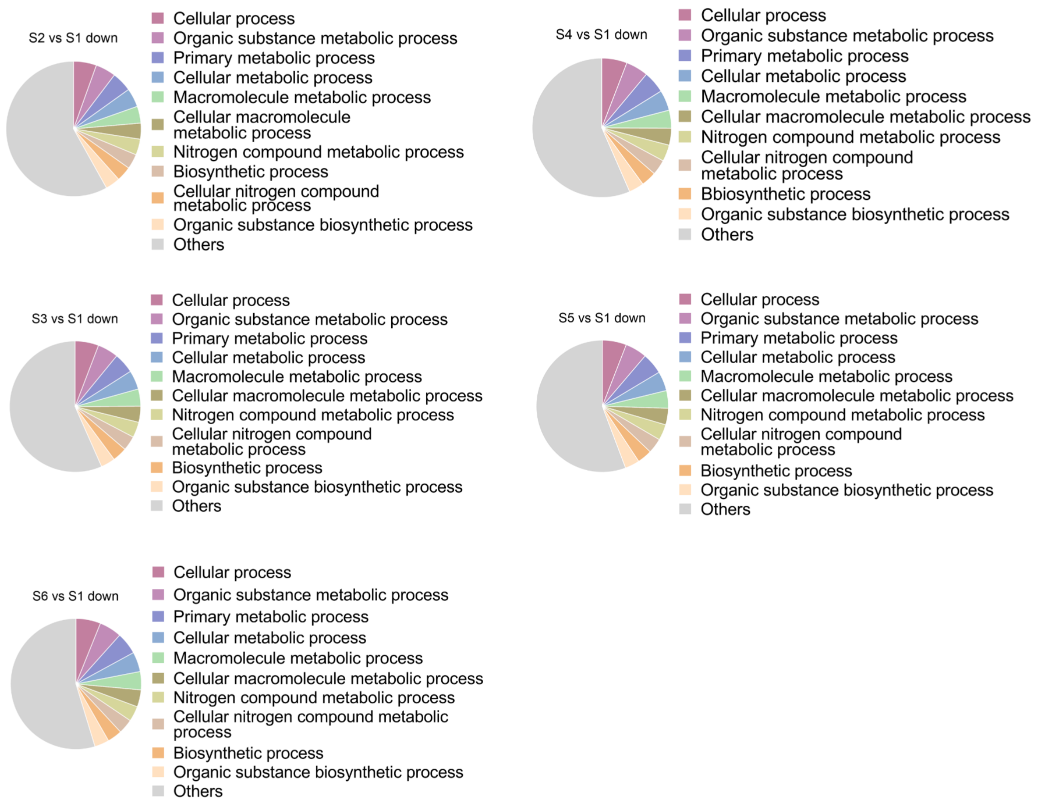

Supplementary Figure 4. Enrichment analysis of down-regulated DEGs during the process of glume-opening and -closing in barley. Gene Ontology analysis of down-regulated DEGs was considered statistically significantly enriched when FDR < 0.05. All Go terms were listed in Supplemental Data Set 1.

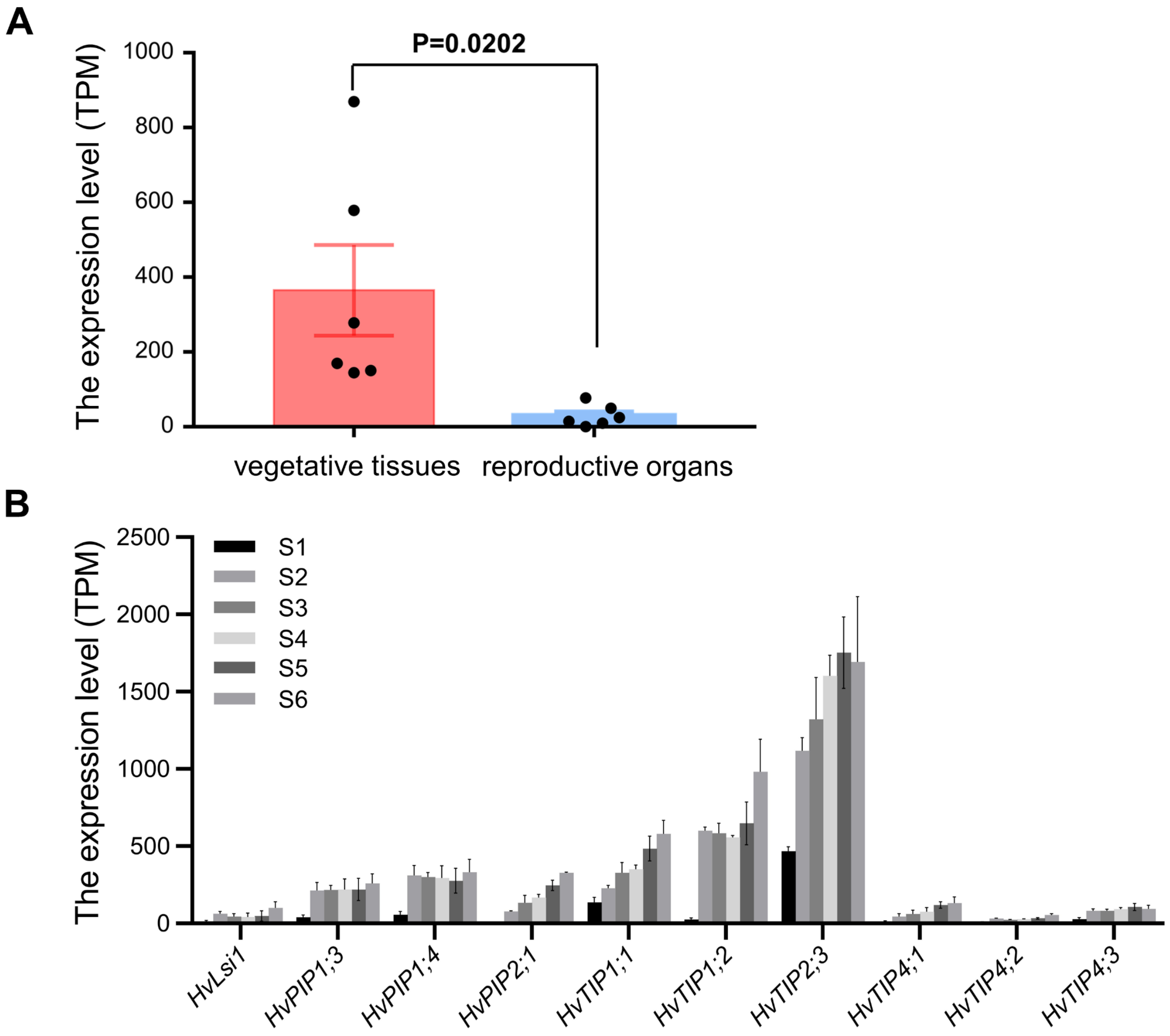

Supplementary Figure 5. Comparison of expression levels of 6 *AQP* genes (*HvTIP4;1*, *HvTIP4;2*, *HvPIP2;2*, *HvPIP2;8*, *HvLsi1*, and *HvLsi6*) in vegetative tissues and reproductive organs (**A**) and the expression levels of 10 differentially expressed *AQP* genes during the process of flowering in barley (**B**). The data are shown as mean ± SD, n = 3. The *P* value indicates the results from student's t test ($P < 0.05$).

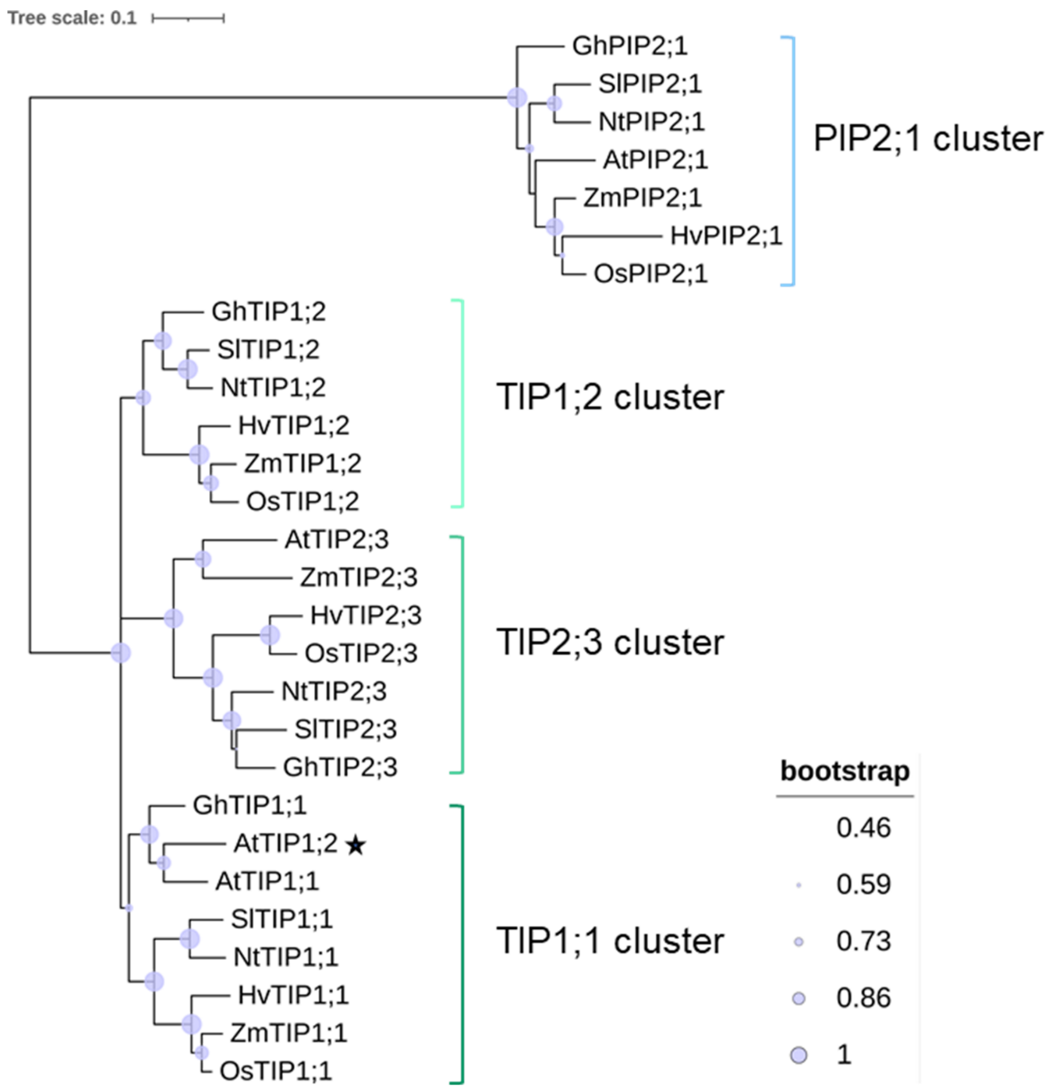

Supplementary Figure 6. Phylogenetic analysis of AQP candidates in *Arabidopsis* and six analysed species. Phylogenetic tree of *TIP1;1*, *TIP1;2*, *TIP2;3* and *PIP2;1* was constructed based on NJ method. The percentages of bootstrap are showed at the branch nodes.

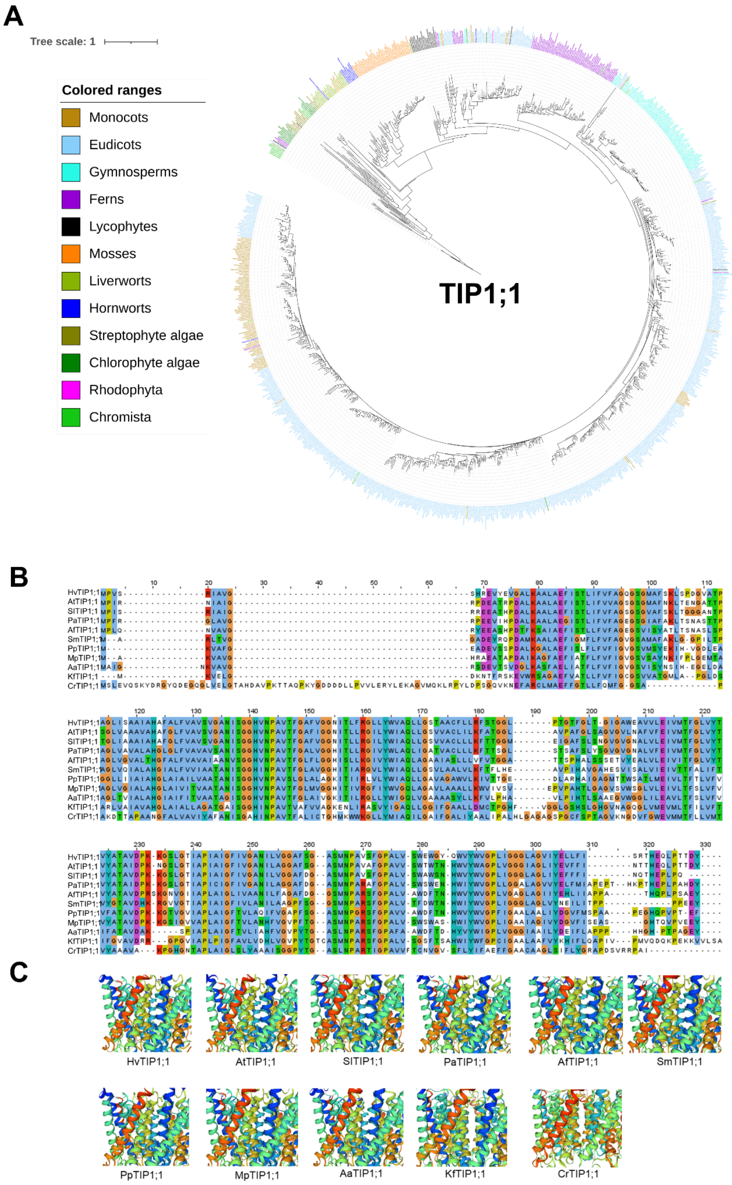

Supplementary Figure 7. Phylogenetic trees, protein sequence alignment and predicted 3D structure of TIP1;1 in plants and algae. (**A**) Phylogenetic trees of TIP1;1 homologs in representative species of major lineage of plants and algae. The maximum likelihood was used to construct the trees. All the transcriptomes can be accessed from the OneKP database. Candidate protein sequences were acquired by BLASTP searches using *HvTIP1;1* as the query with the criterion of E-value < 10^{-5}. (**B**) Protein sequence alignment and (**C**) predicted 3D structure of TIP1;1 in eleven representative plants and algae. The corresponding full Latin name of each species is shown in **Figure 5**.

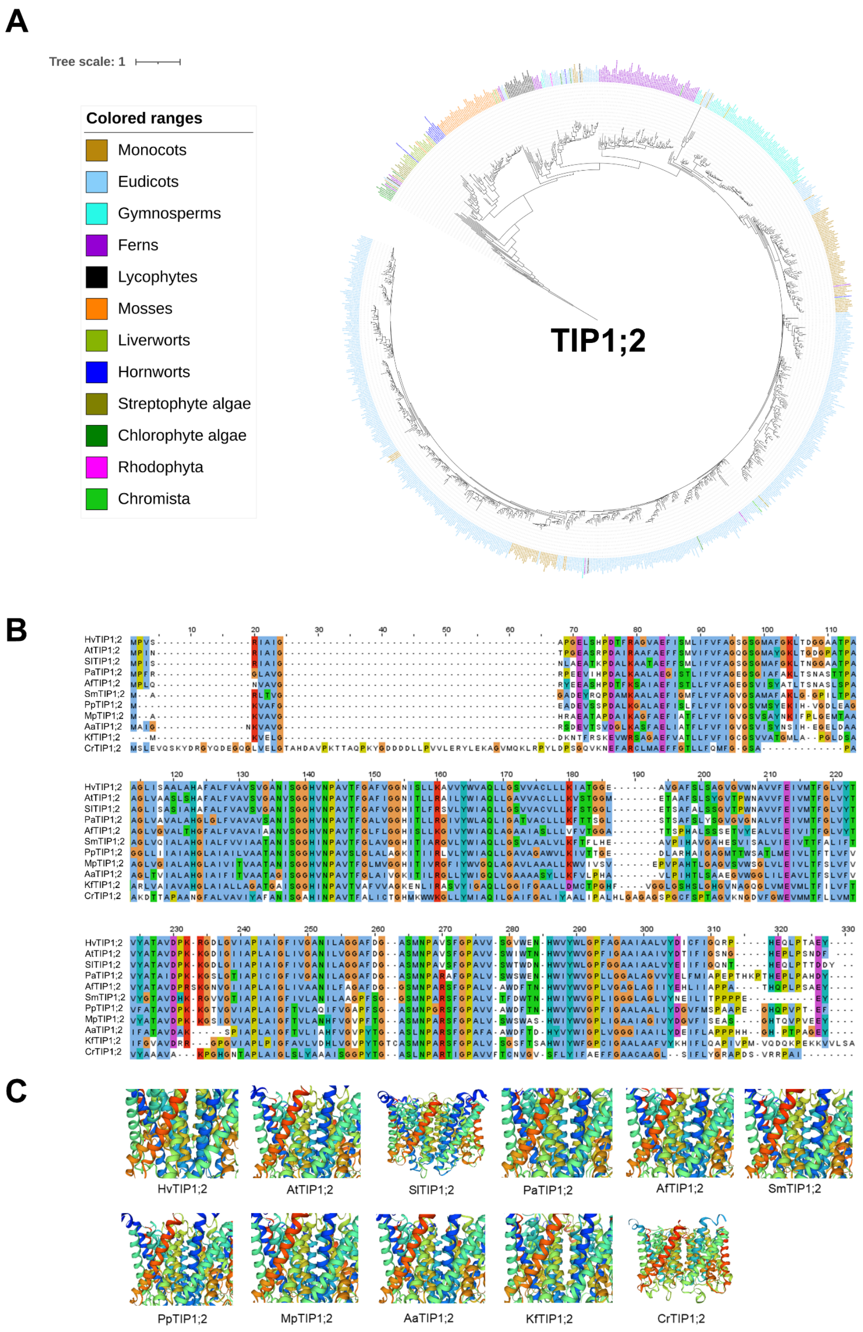

Supplementary Figure 8. Phylogenetic tree, protein sequence alignment and predicted 3D structure of TIP1;2 in plants and algae. (**A**) Phylogenetic tree of TIP1;2 homologs in representative species of major lineage of plants and algae. The maximum likelihood was used to construct the tree. All the transcriptomes can be accessed from the OneKP database. Candidate protein sequences were acquired by BLASTP searches using *HvTIP1;2* as the query with the criterion of E-value < 10^{-5}. (**B**) Protein sequence alignment and (**C**) predicted 3D structure of TIP1;2 in eleven representative plants and algae. The corresponding full Latin name of each species is shown in **Figure 5**.

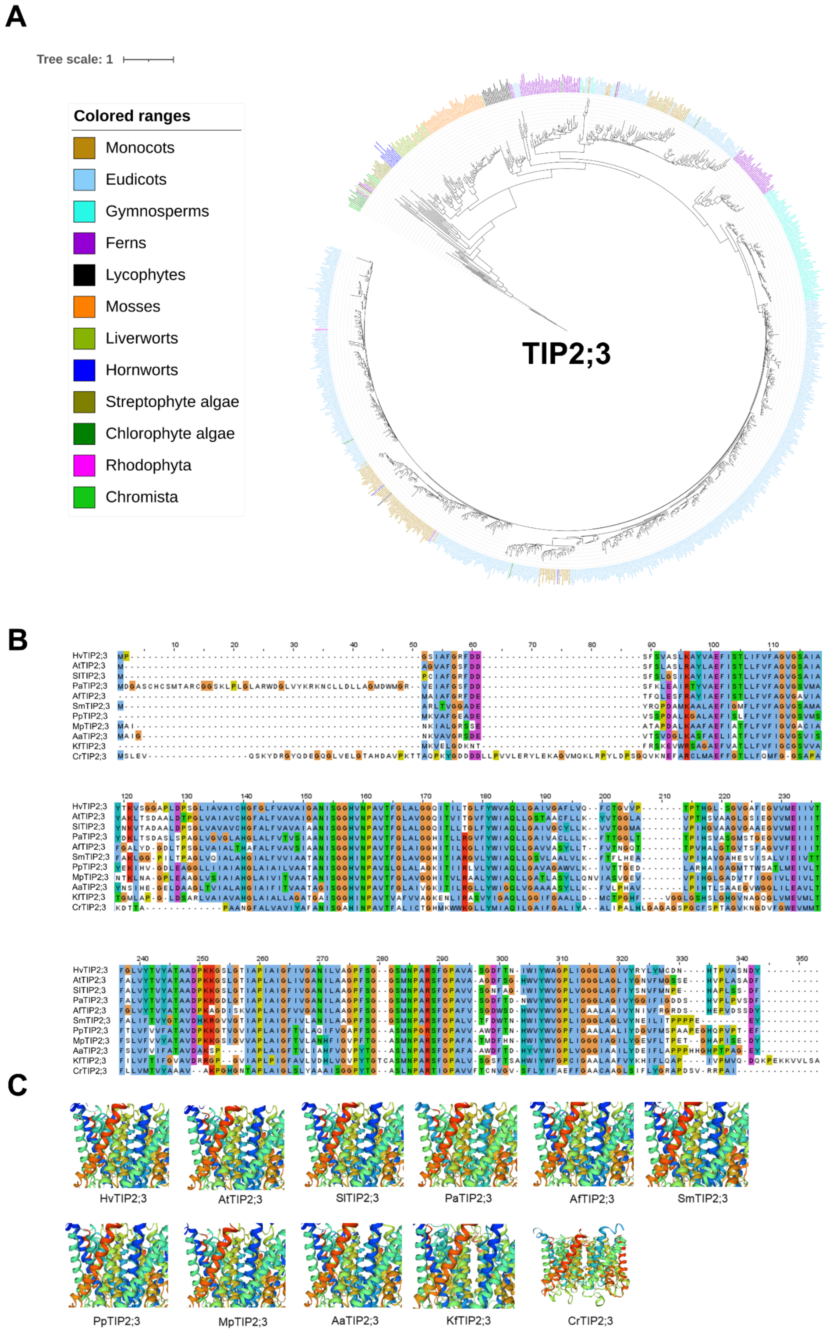

Supplementary Figure 9. Phylogenetic tree, protein sequence alignment and predicted 3D structure of TIP2;3 in plants and algae. (**A**) Phylogenetic tree of TIP2;3 homologs in representative species of major lineage of plants and algae. The maximum likelihood was used to construct the tree. All the transcriptomes can be accessed from the OneKP database. Candidate protein sequences were acquired by BLASTP searches using *HvTIP2;3* as the query with the criterion of E-value < 10^{-5}. (**B**) Protein sequence alignment and (**C**) predicted 3D structure of TIP2;3 in eleven representative plants and algae. The corresponding full Latin name of each species is shown in **Figure 5**.

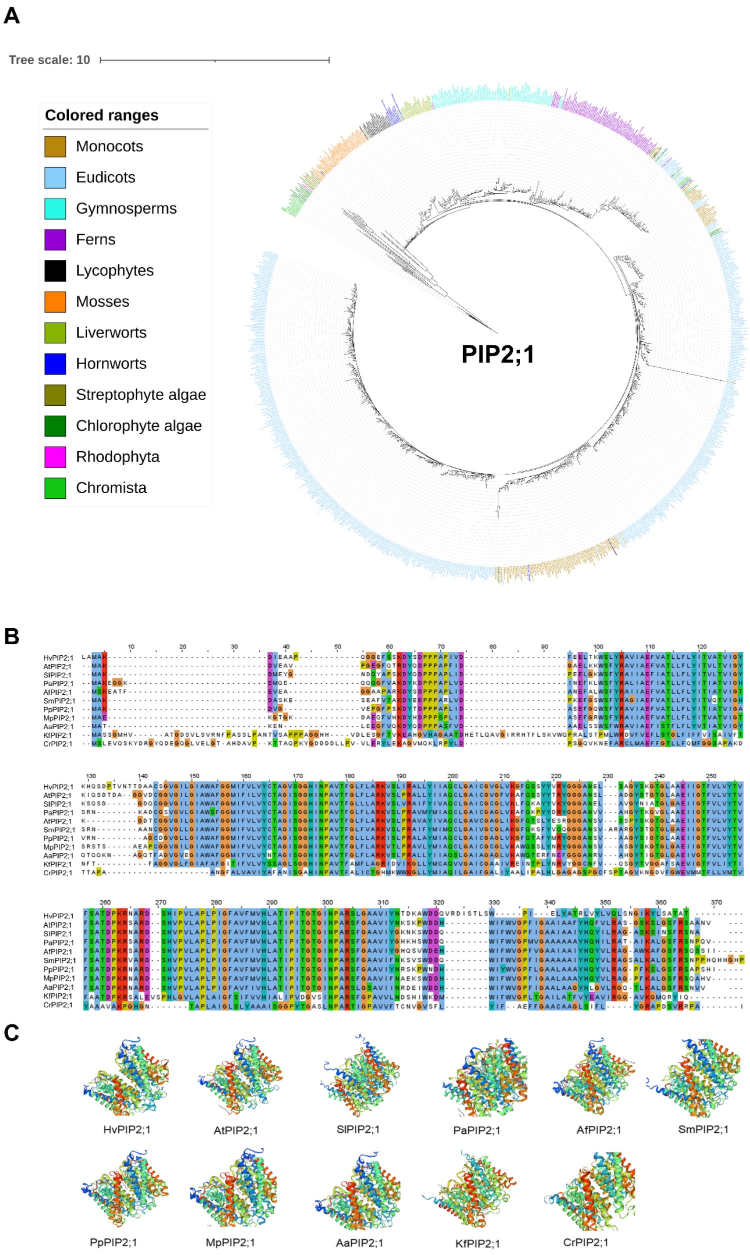

Supplementary Figure 10. Phylogenetic tree, protein sequence alignment and predicted 3D structure of PIP2;1 in plants and algae. (**A**) Phylogenetic tree of PIP2;1 homologs in representative species of major lineage of plants and algae. The maximum likelihood was used to construct the tree. All the transcriptomes can be accessed from the OneKP database. Candidate protein sequences were acquired by BLASTP searches using *Hv PIP2;1* as the query with the criterion of E-value < 10^{-5}. (**B**) Protein sequence alignment and (**C**) predicted 3D structure of PIP2;1 in eleven representative plants and algae. The corresponding full Latin name of each species is shown in **Figure 5**.

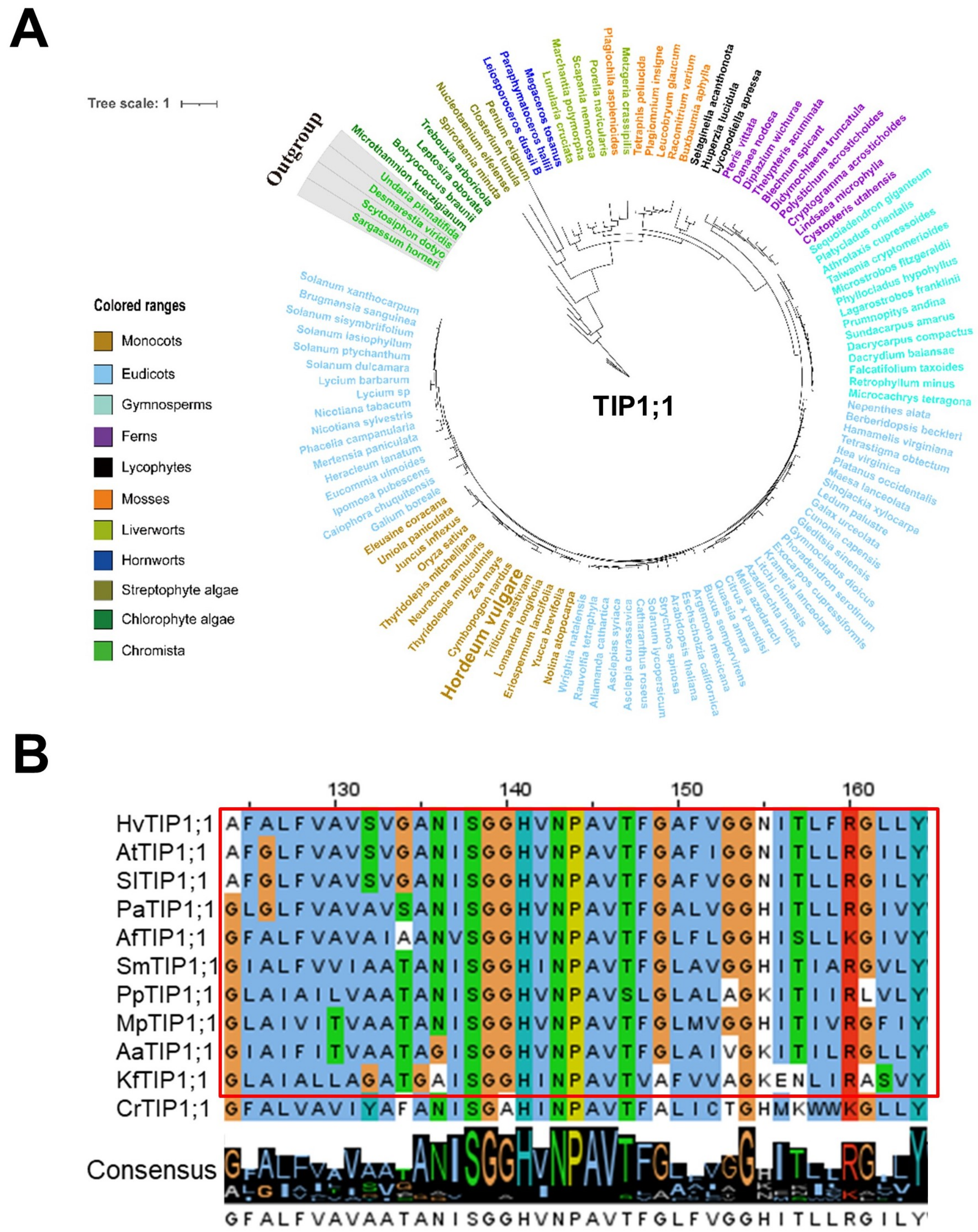

Supplementary Figure 11. Molecular evolutionary analysis of the aquaporins TIP1;1 in plants and algae. (**A**) Phylogenetic tree of TIP1;1 proteins in representative species of major lineage of plants and algae (See **Supplementary Figure 7A** for all OneKP species). The maximum likelihood (ML) method was used to construct the tree. Clades are indicated by different colors. (**B**) Conserved motif alignment of TIP1;1 in eleven representative plants and algal species. The corresponding full Latin name of each species is shown in **Figure 5**.

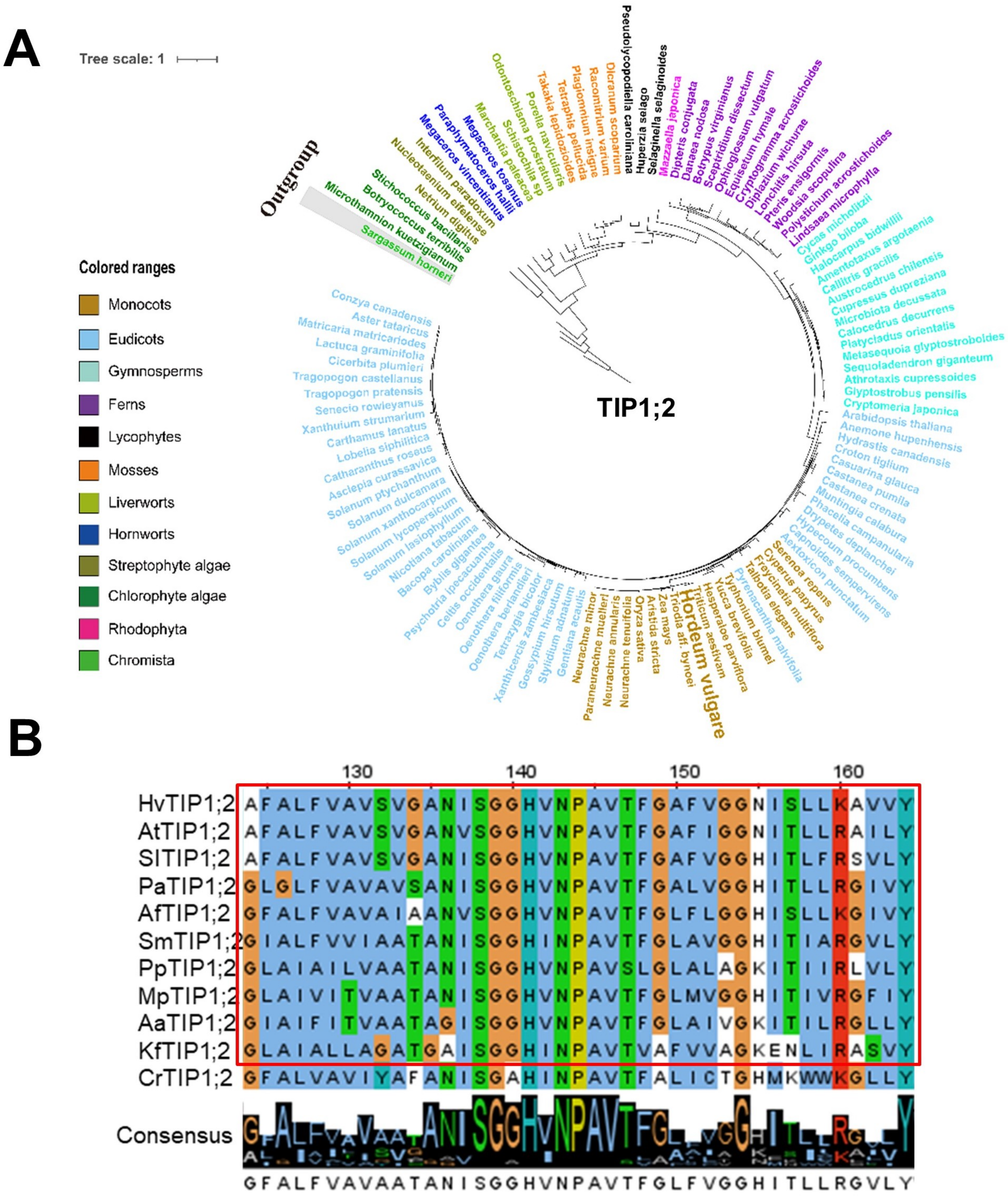

Supplementary Figure 12. Molecular evolutionary analysis of the aquaporins TIP1;2 in plants and algae. (**A**) Phylogenetic tree of TIP1;2 proteins in representative species of major lineage of plants and algae (See **Supplementary Figure 8A** for all OneKP species). The maximum likelihood (ML) method was used to construct the tree. Clades are indicated by different colors. (**B**) Conserved motif alignment of TIP1;2 in eleven representative plants and algal species. The corresponding full Latin name of each species is shown in **Figure 5**.

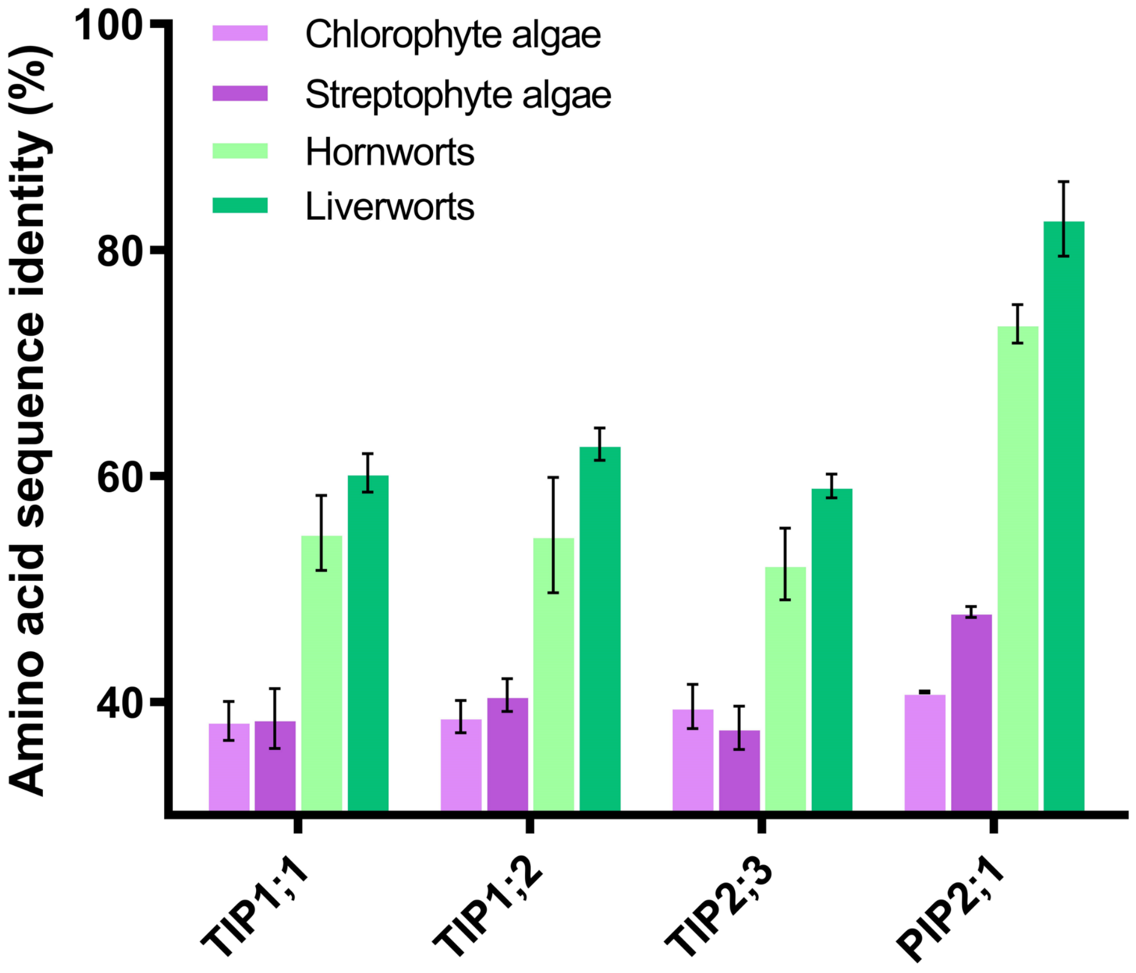

Supplementary Figure 13. The Amino acid sequence similarity comparison of PIP and TIPs in algae and liverworts or hornworts. The data are shown as mean ± SD.

1.2 Supplementary Tables

Supplementary Table 1 Primer sequences used for RT-qPCR.

Primer name	Primer Sequence
HvTIP1.1-F	TCTCCACGCTCATCTTCGTC
HvTIP1.1-R	GGAAGAGGGTGATGTTGCCG
HvTIP1.2-F	CGTCGCCGAGTTCATATCCA
HvTIP1.2-R	GCCTTGAGGAGGCTGATGTT
HvTIP2.3-F	TGGTACCGACCCCTACACAC
HvTIP2.3-R	ATATTGGTGAAGTCGCCGCT
HvPIP2.1-F	AGCTCCTACTACGTGCGGTA
HvPIP2.1-R	GATGTCTCGTACTTGGTCATCC
HvActin-F	GACTCTGGTCATGGTGTCAGC
HvActin-R	GGCTGGAAGAGGACCTCAGG

Supplementary Table 2 The sequence information of six species for absolute expression

Species	Gene	ID	Forward primer (5' → 3')	Reverse primer (5' → 3')	Linear regression equations for standard curves	Coefficient of determination (R^2)
Gossypium hirsutum	*TIP1;1*	*NM_001326874.1*	TCATCAGCAACACCCACGAGCAG	GAAAGAGATAGGAGACTCAAATG	y = -3.3903x + 33.939	0.9975
	TIP1;2	*XM_016895058.1*	GCAGTCTCCTTCGGACCAGCCGT	TCTGGGGCGATGAAGAAGACCTC	y = -3.2513x + 35.717	0.9981
	TIP2;3	*NM_001327578.1*	TATTCAAGGAGTGGTGATGGAGA	CAATGAAGCCGATGGCAATGGGT	y = -3.2707x + 32.695	0.9975
	PIP2;1	*XM_016894983.1*	CCACTCCCCATTGGATTCGCTGT	GCTCTGAGGATGTATTGGTGGTA	y = -3.0103x + 31.549	0.997
Oryza sativa	*TIP1;1*	*XM_015775609.2*	TCCAACCGTTTAGTTTCTCGCAT	ACGGGTTCCCAAAGGTCCACACT	y = -2.943x + 33.207	0.9939
	TIP1;2	*XM_015785670.2*	TCTGCTCCCAGTCTCAATAATCG	ATGAGACAACCGATGGATGGACG	y = -2.923x + 26.382	0.9999
	TIP2;3	*XM_015788427.1*	CAGTGTGGAGCAAGTTGGATGAG	GGCTACAAACAAACTGAAATGAC	y = -3.488x + 31.694	0.9954
	PIP2;1	*XM_015770913.1*	ATGATGAGGCTAAGTCGGTTGTG	ATGGCTGGTTTAGGGATTGATTA	y = -3.2267x + 33.695	0.9998
Zea mays	*TIP1;1*	*NM_001111426.2*	CACCGACTACTAAAAGCCGAAGC	ATTCACGGTTACAAGCAGTTCAG	y = -3.1053x + 30.951	0.9999
	TIP1;2	*NM_001111559.1*	TGGACCCCAAGAAGGGCGACCTC	TATATATTGAGTGGTCTGGTCTT	y = -3.1303x + 31.556	0.9851
	TIP2;3	*XM_008670832.4*	TACTCGCAACTGACAAAGGGTGG	GCCTGTCCGTGGGTGACGAACTG	y = -3.282x + 33.777	0.9994
	PIP2;1	*NM_001111556.1*	GGAGCAACGCCTAAGTTCCGCCC	AGATATGTCACACTTTGGTCCGA	y = -3.0533x + 31.935	0.993
Nicotiana tabacum	*TIP1;1*	*NM_001325942.1*	TCACTTTGTTCCGTGGCATTCTC	CAGTGTAAACAAGTCCAAAAGTC	y = -2.999x + 35.496	0.9977
	TIP1;2	*XM_016646225.1*	TGCTGGATTGATTTCGGCGGCTA	GACCTCCCACGAAAGCACCGAAT	y = -2.6427x + 30.97	0.9939
	TIP2;3	*NM_001325717.1*	TATCTCCACCTTGCTCTTTGTCT	AACGGCTACGAATAGACCAAATC	y = -3.336x + 34.906	0.9947
	PIP2;1	*NM_001324836.1*	AGGGATTCCAGAGTGCTTATTAT	TGGAAGTGGTGCCAACACAGGGA	y = -2.939x + 32.138	0.9965
Solanum lycopersicum	*TIP1;1*	*NM_001247174.2*	TCGCAGGTCAGGGTTCTGGTATG	CGGCGACGAACAACCCGAAGGCG	y = -3.399x + 34.37	0.9952
	TIP1;2	*NM_001287766.1*	CCCTTTTTGTGGCAGTTTCAGTT	GGAGCACACAAGCAACGACGGAT	y = -3.039x + 34.238	0.9886
	TIP2;3	*NM_001302916.1*	ACGGTGTGGCTGCTGGAGTAGGA	ACCACAGCGGGTCCAAATGAACG	y = -2.8723x + 30.732	0.985
	PIP2;1	*AB845612.1*	CCCATTGGATTTGCCGTATTCAT	GGGTAGACAGCGGCGATGAAAGC	y = -2.963x + 32.498	0.9993
Hordeum vulgare	*TIP1;1*	*HORVU4Hr1G079230.1*	CGCTGTTCGTGGCGGTGTCGGTG	TGGAGAAGCGGAGGAGGAAGCAG	y = -3.3017x + 34.786	0.9976
	TIP1;2	*HORVU3Hr1G116790.2*	AGATCGCCACTGGCGGCGAGGC	TACCCCGAGGTCGCCGCGCTTG	y = -2.9283x + 32.182	0.9998
	TIP2;3	*HORVU7Hr1G081770.8*	CGACCCCTACACACGGGCTTTCC	GTTGCTGGCGACGGGGGTGTGGT	y = -2.923x + 33.698	0.9837
	PIP2;1	*HORVU6Hr1G058930.20*	GTGACCTTCGGGTTGTTCCTGGC	CGGTGCCCTTGGAGTAGCCTGCG	y = -2.9093x + 30.981	0.98

Supplementary Table 3 Nomenclature and protein properties of HvAQPs

Subfamily	Gene	Accession ID	AA Length	MW (kDa)	PI	TMD	Subcellular Localization
**PIP**	HvPIP1;1	HORVU2Hr1G096360.13	291	31.2289	9.64	5	PM
	HvPIP1;2	HORVU5Hr1G055200.4	311	32.8355	8.57	6	PM
	HvPIP1;3	HORVU6Hr1G092960.5	292	30.8494	8.3	6	PM
	HvPIP1;4	HORVU6Hr1G092970.3	247	25.6903	7.38	5	PM
	HvPIP1;5	HORVU6Hr1G064140.1	290	30.8535	9.06	6	PM
	HvPIP2;1	HORVU6Hr1G058930.20	289	30.7322	6.92	5	PM
	HvPIP2;10	HORVU6Hr1G014300.1	292	30.6961	9.1	6	PM
	HvPIP2;11	HORVU7Hr1G038940.1	204	21.3794	6.93	3	PM
	HvPIP2;2	HORVU2Hr1G010990.2	318	33.3853	9.91	5	PM
	HvPIP2;3	HORVU2Hr1G089940.5	290	30.4869	7.96	6	PM
	HvPIP2;4	HORVU2Hr1G089820.6	291	30.4869	7.96	6	PM
	HvPIP2;5	HORVU2Hr1G038740.1	341	36.0575	9	6	PM
	HvPIP2;6	HORVU5Hr1G027240.4	318	33.1769	6.5	6	PM
	HvPIP2;7	HORVU5Hr1G029550.2	318	33.1909	6.5	6	PM
	HvPIP2;8	HORVU5Hr1G084230.1	311	32.5976	9.58	6	PM
	HvPIP2;9	HORVU5Hr1G125600.1	291	30.5092	8.81	6	PM
**TIP**	HvTIP1;1	HORVU4Hr1G079230.1	250	25.7697	6.51	6	vacu
	HvTIP1;2	HORVU3Hr1G116790.2	252	25.5164	5.48	7	vacu
	HvTIP2;1	HORVU6Hr1G062980.1	249	25.205	5.96	6	vacu
	HvTIP2;3	HORVU7Hr1G081770.8	249	25.3282	6.06	6	vacu
	HvTIP3;1	HORVU1Hr1G043890.4	263	27.5087	8.03	6	vacu
	HvTIP4;1	HORVU4Hr1G085250.2	301	31.3524	6.71	6	vacu
	HvTIP4;2	HORVU3Hr1G031680.1	268	28.2718	12.8	3	PM
	HvTIP4;3	HORVU3Hr1G031620.5	318	34.0666	12.1	6	vacu
	HvTIP5;1	HORVU2Hr1G097780.15	263	26.904	7.95	6	vacu
**NIP**	HvLsi1	HORVU7Hr1G038270.3	366	40.5969	9.26	4	PM
	HvLsi6	HORVU6Hr1G075850.4	318	34.0312	9.28	5	PM
	HvNIP1;1	HORVU7Hr1G043590.2	309	33.1151	8.67	6	PM
	HvNIP1;3	HORVU5Hr1G085710.4	333	35.3432	8.62	5	PM
	HvNIP1;3a	HORVU7Hr1G121250.3	292	30.596	8.33	5	PM
	HvNIP1;4	HORVU7Hr1G088900.1	211	22.7075	7.84	2	PM
	HvNIP2;2	HORVU7Hr1G038220.1	300	32.2511	8.01	6	PM
	HvNIP3;1	HORVU1Hr1G047100.3	367	37.9562	7.91	5	PM
	HvNIP3;1a	HORVU3Hr1G014440.3	258	27.1072	9.97	6	PM
	HvNIP3;2	HORVU3Hr1G079560.5	247	25.8001	7.03	6	PM
	HvNIP4;1	HORVU3Hr1G001320.1	277	30.376	12.03	4	PM
**SIP**	HvSIP1;1	HORVU4Hr1G024470.2	245	25.4566	8.6	6	PM
	HvSIP2;1	HORVU4Hr1G052170.25	249	26.6564	10.41	6	PM /vacu

PM: plasma membrane; Vacu: vacuolar membrane; AA: Amino acid; MW: molecular weight; pI: isoelectric point; TMD: transmembrane domain. The MW and pI of the amino acid sequences were predicted using the perl script. The TMD prediction was studied using TMHMM Server v .2.0 (http://www.cbs.dtu.dk/services/TMHMM/), and Subcellular localization was analyzed by Plant-mPloc server (http://www.csbio. sjtu.edu.cn/bioinf/plant-multi/).

Supplementary Table 4 DEGs of aquaporin gene family during the floret opening.

	S2 vs S1	FDR	S3 vs S1	FDR	S4 vs S1	FDR	S5 vs S1	FDR	S6 vs S1	FDR
HvTIP1;1	0.74	4.66E-03	1.26	8.46E-06	1.36	2.31E-09	1.82	6.70E-13	2.08	3.95E-16
HvTIP1;2	4.52	1.04E-79	4.48	3.82E-60	4.42	2.21E-72	4.64	3.18E-50	5.23	6.60E-58
HvTIP2;3	1.26	5.46E-24	1.5	1.68E-13	1.78	2.98E-48	1.91	2.10E-33	1.86	2.20E-16
HvTIP4;1	1.86	3.99E-03	2.33	1.18E-04	2.67	5.08E-07	3.31	4.63E-15	3.45	7.62E-14
HvTIP4;2	2.12	6.36E-04	1.72	1.24E-02	1.91	4.65E-03	2.23	4.57E-04	2.91	5.48E-08
HvTIP4;3	1.5	9.79E-05	1.5	7.83E-05	1.68	3.94E-06	1.88	1.59E-06	1.7	1.15E-05
HvPIP1;3	2.36	3.40E-13	2.39	2.76E-16	2.42	2.87E-11	2.42	7.11E-11	2.65	4.69E-16
HvPIP1;4	2.43	1.08E-15	2.38	6.26E-17	2.35	7.54E-12	2.26	1.85E-10	2.53	4.98E-14
HvPIP2;1	4.43	5.65E-16	5.19	1.66E-18	5.53	1.76E-27	6.08	3.05E-33	6.48	3.15E-41
HvLsi1	2.38	1.03E-05	1.86	8.51E-03	1.83	1.54E-02	2.01	1.05E-02	3.04	9.99E-08

Supplementary Table 5 Statistics of evolution of TIP1;1, TIP1;2, TIP2;3 and PIP2;1 in the 1KP dataset.

order	Total No, of species	TIP1;1 in No, of species	TIP1;2 in No, of species	TIP2;3 in No, of species	PIP2;1 in No, of species	TIP1;1%	TIP1;2%	TIP2;3%	PIP2;1%
Monocots	116	106	104	105	106	91	90	91	91
Eudicots	584	558	544	539	559	96	93	92	96
Gymnosperms	84	80	79	77	80	95	94	92	95
Ferns	88	74	74	73	74	84	84	83	84
Lycophytes	22	21	20	20	21	95	91	91	95
Mosses	52	42	42	44	42	81	81	85	81
Liverworts	30	25	25	24	24	83	83	80	80
Hornworts	15	11	11	11	10	73	73	73	67
Streptophyte algae	48	17	11	16	9	35	23	33	19
Chlorophyte algae	121	18	12	12	19	15	10	10	16
Rhodophyta	34	1	1	1	1	3	3	3	3
Chromista	37	11	3	8	15	30	8	22	41
Total/Average	1231	964	926	930	960	78	75	76	78

Note: Candidate protein sequences were selected by BLASTP searches using *Hordeum vulgare* sequences as the query with the criterion of E-value < 10^{-5} in the 1KP database.
